# FOXO1 reshapes neutrophils to aggravate acute brain damage and promote late depression after traumatic brain injury

**DOI:** 10.1186/s40779-024-00523-w

**Published:** 2024-03-31

**Authors:** Mi Zhou, Yang-Wu-Yue Liu, Yu-Hang He, Jing-Yu Zhang, Hao Guo, Hao Wang, Jia-Kui Ren, Yi-Xun Su, Teng Yang, Jia-Bo Li, Wen-Hui He, Peng-Jiao Ma, Man-Tian Mi, Shuang-Shuang Dai

**Affiliations:** 1 Department of Biochemistry and Molecular Biology, School of Basic Medicine, Army Medical University, Chongqing, 400038 China; 2 Research Center for Nutrition and Food Safety, Chongqing Key Laboratory of Nutrition and Health, Institute of Military Preventive Medicine, Army Medical University, Chongqing, 400038 China; 3 Department of Neurosurgery, Daping Hospital, Army Medical University, Chongqing, 400042 China; 4 Department of Trauma and Emergency, Southwest Hospital, Army Medical University, Chongqing, 400038 China; 5 Department of Histology and Embryology, Chongqing Key Laboratory of Neurobiology, Brain and Intelligence Research Key Suyixun Laboratory of Chongqing Education Commission, Army Medical University, Chongqing, 400038 China; 6 Research Center, Seventh Affiliated Hospital of Sun Yat-Sen University, Shenzhen, 518107 Guangdong China

**Keywords:** Traumatic brain injury (TBI), Neutrophil, Forkhead box protein O1 (FOXO1), Acute stage, Chronic stage

## Abstract

**Background:**

Neutrophils are traditionally viewed as first responders but have a short onset of action in response to traumatic brain injury (TBI). However, the heterogeneity, multifunctionality, and time-dependent modulation of brain damage and outcome mediated by neutrophils after TBI remain poorly understood.

**Methods:**

Using the combined single-cell transcriptomics, metabolomics, and proteomics analysis from TBI patients and the TBI mouse model, we investigate a novel neutrophil phenotype and its associated effects on TBI outcome by neurological deficit scoring and behavioral tests. We also characterized the underlying mechanisms both in vitro and in vivo through molecular simulations, signaling detections, gene expression regulation assessments [including dual-luciferase reporter and chromatin immunoprecipitation (ChIP) assays], primary cultures or co-cultures of neutrophils and oligodendrocytes, intracellular iron, and lipid hydroperoxide concentration measurements, as well as forkhead box protein O1 (FOXO1) conditional knockout mice.

**Results:**

We identified that high expression of the FOXO1 protein was induced in neutrophils after TBI both in TBI patients and the TBI mouse model. Infiltration of these FOXO1^high^ neutrophils in the brain was detected not only in the acute phase but also in the chronic phase post-TBI, aggravating acute brain inflammatory damage and promoting late TBI-induced depression. In the acute stage, FOXO1 upregulated cytoplasmic Versican (VCAN) to interact with the apoptosis regulator B-cell lymphoma-2 (BCL-2)-associated X protein (BAX), suppressing the mitochondrial translocation of BAX, which mediated the antiapoptotic effect companied with enhancing interleukin-6 (IL-6) production of FOXO1^high^ neutrophils. In the chronic stage, the “FOXO1-transferrin receptor (TFRC)” mechanism contributes to FOXO1^high^ neutrophil ferroptosis, disturbing the iron homeostasis of oligodendrocytes and inducing a reduction in myelin basic protein, which contributes to the progression of late depression after TBI.

**Conclusions:**

FOXO1^high^ neutrophils represent a novel neutrophil phenotype that emerges in response to acute and chronic TBI, which provides insight into the heterogeneity, reprogramming activity, and versatility of neutrophils in TBI.

**Supplementary Information:**

The online version contains supplementary material available at 10.1186/s40779-024-00523-w.

## Background

Traumatic brain injury (TBI) is a public health issue that ranks among the primary causes of both mortality and disability worldwide, even though treatments have significantly improved in recent years [1, 2]. It is a complex process involving a broad spectrum of symptoms and long-term consequences. Generally, the pathobiology of TBI is composed of two parts: primary injury and secondary injury [3]. The primary injury is the mechanical impact that initiates a cascade of acute brain damage, including skull fractures, hemorrhage, and neuron death. Conversely, the secondary injury triggers a robust immune response along with the toxic effects caused by an abrupt surge in intracellular calcium and an extensive release of excitatory amino acids, leading to further ischemia, brain swelling, and intracranial hypertension. The outcomes following TBI are not only determined by the severity of the primary injury but also influenced by the secondary injury, which may result in acute neurological impairments as well as long-term chronic functional, neurocognitive, and neuropsychiatric deficits [4]. Accordingly, the identification of common underlying factors or key regulatory mechanisms in both acute and chronic damages post-TBI is crucial for the development of treatment protocols for TBI.

Notably, immune-based inflammation is a major secondary injury mechanism that contributes to acute neuronal cell damage or death following TBI and is also implicated in chronic brain dysfunction in the repair stage post-TBI [5]. In addition to brain-resident inflammatory cells such as microglia and astrocytes, peripheral immune cells are also activated and infiltrate the brain because of the compromised blood–brain barrier (BBB). Neutrophils, the most prevalent leukocytes in peripheral immune cells, are the first-line defenders infiltrating the brain for pathogen and debris clearance after TBI and are also the initiating factor of neuroinflammation [6, 7]. Traditionally, neutrophils are believed to be short-lived immune cells and only affect the acute stage of TBI. However, increasing evidence demonstrated that neutrophils exacerbate tissue damage in chronic inflammatory diseases through different mechanisms. For example, neutrophils secrete proteases and further alter interleukin (IL)-22 receptor-dependent signalling to enhance pathogen replication and ultimately exacerbate chronic obstructive pulmonary disease [8]. In addition, neutrophil extracellular traps induce intestinal damage and thrombotic tendency in inflammatory bowel disease [9]. Furthermore, a higher frequency of neutrophils in circulation is strongly associated with liver enzymes, grade of steatosis, inflammation and fibrosis, hepatocellular ballooning, and nonalcoholic fatty liver disease activity score, which contribute to reducing immunological defence and further accelerating the progression of hepatic steatosis [10]. However, it is largely unknown whether neutrophils are also essential in the regulation of chronic brain damage and consequences in the chronic stage of TBI besides their important acute response and modulation post-TBI. Among these deficits in the chronic stage, depression is one of the most common complications and its incidence rate is as high as 30–77% within 1–50 years after injury, which has become one of the biggest bottlenecks severely restricting the cure of TBI [6]. Growing evidence showed that peripheral and central immune factors like tumor necrosis factor (TNF)-α, IL-1 and IL-6 significantly increased in both animals and patients of depression after TBI, and inflammatory signaling in animals plays an integral role in depressive-like behaviors after brain injury [6, 11–13]. Since neutrophil is the major source of these inflammatory cytokines and has also been found to exert effects in some chronic medical conditions, investigation of its roles and underlying mechanism in acute and chronic phases of TBI will provide insights into the ability of neutrophils in TBI and expand our view about the neuroinflammation-associated chronic disorders such as depression post-TBI.

Forkhead box protein O1 (FOXO1), a mammalian evolutionarily conserved transcription factor, is involved in biological activities ranging from the cell cycle and apoptosis to stress responses. FOXO1 is gradually being noticed in the homeostasis of immune-relevant cells [14], including lymphocytes and macrophages, in a variety of diseases, such as arthritis and systemic lupus erythematosus. For instance, FOXO1 regulates L-selectin CD62L expression and inhibits late-stage natural killer (NK) cell maturation, thus promoting NK cell homing to lymph nodes [15]. FOXO1 positively regulates major histocompatibility complex II genes in macrophages, promotes M2 polarization, and enhances tumor growth [16]. In hepatic ischemia–reperfusion injury, FOXO1 reduces the polarization of T helper 17 cells to alleviate ischemia–reperfusion stress [17]. Here, using single-cell RNA sequencing (scRNA-seq), it was observed that a subset of neutrophils characterized by elevated levels of FOXO1 remains consistently present during both the acute and chronic stages after TBI. Therefore, an examination was conducted to explore the implications of prolonged FOXO1^high^ neutrophils and its associated role and mechanism in the regulation of brain function after TBI. Given the lack of success in developing effective immunomodulatory therapies for clinical TBI treatment, this study aims to provide new evidence and innovative therapeutic strategies focused on regulating neutrophil-associated immune responses in TBI.

## Materials and methods

### TBI patients

Twenty-two subjects (11 healthy donors and 11 TBI patients) were involved and had no previous history of brain injury. Peripheral blood was collected from healthy donors and TBI patients in ethylene diamine tetraacetic acid-coated tubes. The TBI-injured brain tissues were obtained from TBI patients during treatment according to the planned surgical procedures for removing the damaged brain tissue. The health donors were from the Physical Examination Center, while the TBI patients were from the Department of Neurosurgery, Daping Hospital (Army Special Medical Center). The patients met specific criteria for inclusion, which included being three male individuals aged between 40 and 70 years, diagnosed with moderate or severe TBI and having a Glasgow Coma Score ranging from 7 to 12 within 48 h of the injury. All procedures were approved by the Institutional Research Ethics Committee of Army Medical University, and written informed consent was given by the relatives of each patient in the Department of Neurosurgery, Daping Hospital (Army Special Medical Center). All samples were deidentified and coded by the attending surgeon before transport to the laboratory. The specimens were acquired and processed based on Clinical Investigation and Ethical Approval from the Daping Hospital (Army Special Medical Center) and stored according to the Principles of Human Sample Preservation in China.

### Mice and TBI model

C57BL/6 WT mice (*n* = 400) and FOXO1^△Lyz2^ mice (*n* = 100) used were 8–10 weeks old and housed in a pathogen-free environment at the animal care center of Army Medical University, China. All procedures used in this study were performed under the ethical approval of the Army Medical University Institutional Animal Care and Use Committee (AMUWEC2019085). C57BL/6 FOXO1^fl/fl^ mice were a kind gift from Pro. Deng Laboratory at the Institute of Materia Medica, College of Pharmacy in Army Medical University. By crossing FOXO1-floxed mice with Lyz2-Cre mice purchased from Cyagen Biosciences of China, which carried the Cre recombinase inserted in the Lysozyme-M (Lyz2) gene locus, we specifically deleted FOXO1 in myeloid cells including neutrophils (named as FOXO1^△Lyz2^) [18].

The mouse model of TBI was constructed as described previously using an automatic impact machine (LinTech, Monrovia, CA, USA) and a downstroke (velocity, 2.5 m/s; deformation depth, 3.5 mm; duration, 150 ms) to create a degree of severe TBI [19, 20]. This reproducible and consistent model is generally associated with 30% mortality within the first 5 min after injury. The sham group underwent the same surgical procedures without impact. The anesthetic recovery time of mice was also assayed to exclude the possibility of anesthesia-induced neurological deficit. To investigate the antagonistic effect of FOXO1 on TBI, we used the selective small molecule inhibitor, 5-amino-7-(cyclohexylamino)-1-ethyl-6-fluoro-4-oxo-1,4-dihydroquinoline-3-carboxylic acid (AS1842856, HY-100596, MCE, USA), which specifically targets FOXO1. The mice were administered intraperitoneal injections of AS1842856 (10 mg/kg) either 1 h before TBI or on day 30 post-TBI to assess its impact on both acute or chronic stages of TBI.

The experimental design and main detection indexes are shown in Additional file 1: Materials and methods and Fig. S1.

### Neutrophil isolation and transfection

Neutrophils from bone marrow and peripheral blood were isolated from mice and humans by Tianjin Haoyang Biological Manufacture Company, China (Lot: LZS11131, LZS1100, TBD2013NR), according to the manufacturer’s protocol. The purity of neutrophils was > 85%, which was confirmed by the fluorescence-activated cell sorting (FACS) with a specific marker [mouse: CD45^+^ (1:200, 103112), CD11b^+^ (1:200, 163702), LY6G^+^ (1:200, 127626); human: CD16^+^ (1:200, 302006), CD66b^+^ (1:200, 305118), CD177^+^ (1:200, 315810)] (Biolegend, USA). The attained neutrophils were diluted in RPMI-1640 media (SH30027.01, HyClone, USA) with 10% (v/v) foetal bovine serum (FBS) (10,091,148, Gibco, USA) to a final concentration and cultured at 37 °C in an atmosphere of 5% (v/v) CO_2_. All siRNAs were obtained from Wuhan GeneCreate Biological Engineering Co., Ltd. The siRNA sequences for the target genes were listed in Additional file 1: Table S1. The different plasmid was transfected by 4D-Nucleofector system (LONZA company, Swiss) using HL-60 mode. After transfection, the neutrophils were quickly diluted in RPMI-1640 media with 20% (v/v) FBS.

### Primary cell culture and co-culture system

Mouse oligodendrocyte progenitor cells (OPCs) were isolated by immunostaining from wild-type (WT) mice as Niu et al. [21] described. The purified OPCs were cultured in poly-D-lysine-coated dishes with OPC mediums in the presence of platelet-derived growth factor AA (PDGF-AA, 10 ng/ml, 100-13A, Peprotech, USA). About 5 d later, the medium was replaced by PDGF-AA free medium to induce OPC differentiates into oligodendrocytes.

Primary neurons were isolated from E18−20 embryos in pregnant mice. The brain cortices were micro-dissected, trypsin digested at 37 ℃, filtered through a 70 µm cell strainer, and cultured onto 6-well plates over poly-D-lysine the surfaces with neurobasal mediums contained with B27 and GlutaMAX for the next 7 d.

On day 7, neutrophils were cultured with primary oligodendrocytes or neurons in 6-well plates for co-culture. The oligodendrocyte-neutrophil medium consisted of 80% OPC medium without PDGF-AA and 20% neutrophil medium. The neuron-neutrophil medium comprised 80% neuron medium and 2% neutrophil medium.

### scRNA-seq and neutrophil subclustering

Immune cells were isolated from peripheral blood using a high-density Ficoll gradient. Briefly, peripheral blood was diluted tenfold with FACS buffer [2% FBS in phosphate-buffered saline (PBS)], carefully layered on a Ficoll gradient (11191, Sigma, Germany) and centrifuged at 400 *g* for 30 min at room temperature. The buffy coat was carefully removed, diluted fivefold with FACS buffer, pelleted (300 *g*, 5 min, 4 °C), and incubated in cold FACS buffer containing DNase *I* (LS006344, Worthington Biochemical Corporation, USA) for 10 min at 4 °C. Clumps were dispersed into a single-cell suspension by gentle pipetting. Single-cell suspensions were prepared and loaded on a Chromium Controller (10 × Genomics, Pleasanton, CA, USA) following the manufacturer’s specifications. The Chromium™ Controller and Chromium™ Single Cell 3′ Reagent Version 2 Kit (10 × Genomics, Pleasanton, CA, USA) were used to construct the single-cell library and sequenced using 10 × scRNA-seq by the DNBSEQ platform (BGI-Shenzhen, China).

The gene count matrix generated from Cell Ranger v7.1.0 was converted into a data format (AnnData) compatible with the Scanpy v1.9.3 [22] platform. Doublets were removed by Scrublet v0.2.1 [23, 24] using the default parameters. The principal component analysis was performed for dimensionality reduction using the scanpy.tl.pca function (svd_solver = “arpack”). Uniform manifold approximation and projection were then used for two-dimensional visualization of the resulting clusters. Marker genes of each cluster were identified using the scanpy.tl.rank_genes_groups function (method = “Wilcoxon”) and the clusters were annotated by cell typist [25]. Differential genes were annotated using Gene Ontology (GO) enrichment analyses.

To determine the subcluster in the neutrophils, Leiden clustering was performed with a resolution of 0.1, and marker genes of each cluster were identified with the Wilcoxon rank-sum test. The measurement of RNA velocity in neutrophils was conducted using scVelo to elucidate the dynamic processes [26]. To determine the biological activities between the subclusters of neutrophils, we employed the decoupler to analyze their biological processes [27]. Then, we used cell phone DB with the default parameters to identify the crosstalk between the subclusters of neutrophil cells [28].

### Liquid chromatography-mass spectrometry (LC‑MS) analysis of cell metabolites

A liquid–liquid extraction and LC-MS method was applied to determine the metabolites in isolated neutrophils. Primarily isolated neutrophils were collected by high-speed centrifugation (13,000 *g*, 20 min) and lyophilized for the following steps by Shanghai Luming Biological Technology Company, China. Each group contained 8 individual samples. In brief, isolated neutrophils were analyzed using an Agilent 7890B gas chromatography system (Agilent Technologies Inc., CA, USA) coupled to an Agilent 5977A Mass Selection Detector system (Agilent Technologies Inc., CA, USA). The data preprocessing and statistical analysis were performed using the Analysis Base File Converter software. Metabolites exhibiting a ford change greater than 1.0 and a *P*-value less than 0.05 were considered as differential metabolites.

### Tandem mass tag proteomics analysis

The isolated neutrophils were transferred to a 1.5 ml centrifuge tube, and subsequently lysed with lysis buffer containing 8 mol/L urea, 100 mmol/L triethylammonium bicarbonate at pH 8.5. Following that, the sample was ultrasonicated for 5 min on ice. The lysate was centrifuged at 12,000 *g* for 15 min at 4 ℃, and the supernatant was subsequently reduced with 10 mmol/L dl-dithiothreitol for 1 h at 56 ℃. This was followed by alkylation using sufficient iodoacetamide for 1 h at room temperature in the dark. Each protein sample underwent labeling with tandem mass tag and subsequent desalting and lyophilization. The sample was fractionated on a Rigol L3000 HPLC system (RIGOL TECHNOLOGIES CO., LTD, China) using a C18 column (Waters BEH C18, 4.6 mm × 250 mm, 5 μm) with the column chamber maintained at a temperature of 45 °C. For the construction of transition libraries, we conducted shotgun proteomic analyses using an EASY-nLCTM 1200 UHPLC system (Thermo Fisher, USA) and a Q ExactiveTM series mass spectrometer (Thermo Fisher, USA) in the data-dependent acquisition mode. The statistical analysis of protein quantitation results was performed using *t*-test. Proteins showing significant different were considered differentially expressed if they exhibited fold changes greater than 1.5 and a *P*-value less than 0.05 [29].

### Western blotting

To investigate the associated protein activation, Western blotting analysis was performed. The protein samples were mixed with sample loading buffer (P0015F, Beyotime, China) and subjected to heat at 95 °C for 5 min before being separated on 10% SDS-PAGE gels, and subsequently transferred onto polyvinylidene fluoride membranes. The used antibodies were: FOXO1 (1:1000, 2880 s, Cell Signaling Technology, USA), VCAN (1:500, ET7107-09, Huabio, China), BAX (1:3000, ET1603-34, Huabio, China), BCL-2 (1:3000, ER0602, Huabio, China), neuronal nuclear antigen (NeuN; 1:1000, 36,662, Cell Signaling Technology, USA), MBP (1:1000, PA1-10,008, Thermo Scientific, USA). Finally, the blots were scanned and analyzed by ImageJ software. The normalized band intensities against the corresponding housekeeping protein β-actin (1:1000, 3700 s, Cell Signaling Technology, USA) were calculated for precise comparison.

### Quantitative real‑time PCR (qRT‑PCR)

Tissues or cells were washed with ice-cold PBS and then lysed directly with TRIzol (15596026, Thermo Fisher, USA). Total RNA was isolated by the RNeasy Plus Mini Kit (73404, Qiagen, Germany), and reverse transcription was carried out using the Reverse Transcriptase kit (DRR047S, Takara, Japan), all according to the manufacturer’s protocol. qPCR was carried out using FastStart Universal SYBR Green Master Mix (04707516001, Roche, Switzerland) with a Real-Time PCR Detection System (Roche, Switzerland). The relative expression of target genes was calculated using the 2^−ΔΔCt^ method normalized to the housekeeping gene β-actin. The results are presented as a relative change compared to the controls. All the primers for qRT‑PCR were listed in Additional file 1: Table S2.

### Molecular docking assay

The VCAN crystal structure was predicted by AlphaFold2, and the BAX crystal structure was based on PDB code: 5w62. The Pymol software was further used to pretreat the proteins. Molecular docking assays were performed by using the GRAMM-X server. After that GROMCS 2020 was used for molecular dynamics simulation, and the AMBER99SB-ILDN force field was selected for 50 ns simulation. Finally, the gmx_MMPBSA package was used to calculate the binding interactions between the two proteins and Pymol software visualizes the results.

### Co-immunoprecipitation (Co-IP)

Co-IP was performed using the Pierce Crosstalk Magnetic IP/Co-IP Kit (88,805, Thermo Fisher Scientific, USA). Neutrophils were isolated at a total of 1 × 10^8^ cells and lysed in lysis buffer supplemented with a protease/phosphatase inhibitor cocktail (Pierce) for 30 min on ice. The lysate was then collected by high-speed centrifugation (13,000 *g*, 10 min) at 4 ℃. The supernatant was incubated with BAX antibody (1:200, ET1603-34, Huabio, China), cross-linked to protein A/G magnetic beads, and incubated overnight in a refrigerator at 4 ℃. After washing with IP lysis buffer, the products underwent Western blotting analysis.

### Measurement of intracellular iron and lipid hydroperoxide (LPO) concentrations

Neutrophils were isolated at a total of 1 × 10^6^ cells. Then the iron concentration was quantified using the Cell Total Iron Colorimetric Assay Kit (E-BC-K880-M, Elabscience, China). The LPO concentration was quantified using a Lipid Peroxide Content Assay Kit (BC5245, Solarbio Life sciences, China). All the procedures are according to the manufacturer’s protocol. The reduction of Fe^3+^ to Fe^2+^ in the presence of the chromogen was quantified by measuring the absorbance at 590 nm and the LPO was measured at 532 nm and 600 nm using a spectrophotometer (Thermo Fisher, USA).

### Cell respiration measurements

Ten thousand neutrophils were prepared to measure the oxygen consumption rate by using the Seahorse XFp Real-Time ATP Rate Assay Kit (Kit 103591-100, Agilent, USA). All the procedures are according to the manufacturer-recommended protocols.

### Statistical analysis

All data were analyzed and presented by using GraphPad Prism Software (version 8). Unpaired two-tailed Student’s* t*-test was applied to compare treated groups with vehicle controls. To analyze parameters depending on two or more factors, two-way ANOVA/multivariate analysis of variance was used with Bonferroni correction. *P* < 0.05 was considered statistically significant. Data are represented as the mean ± SEM with ^*^P < 0.05, ^**^P < 0.01, ^***^P < 0.001.

## Results

### Multilevel omics integration reveals high expression of FOXO1 and its associated target gene profile in neutrophils in the acute stage following TBI

**Fig. 1:**
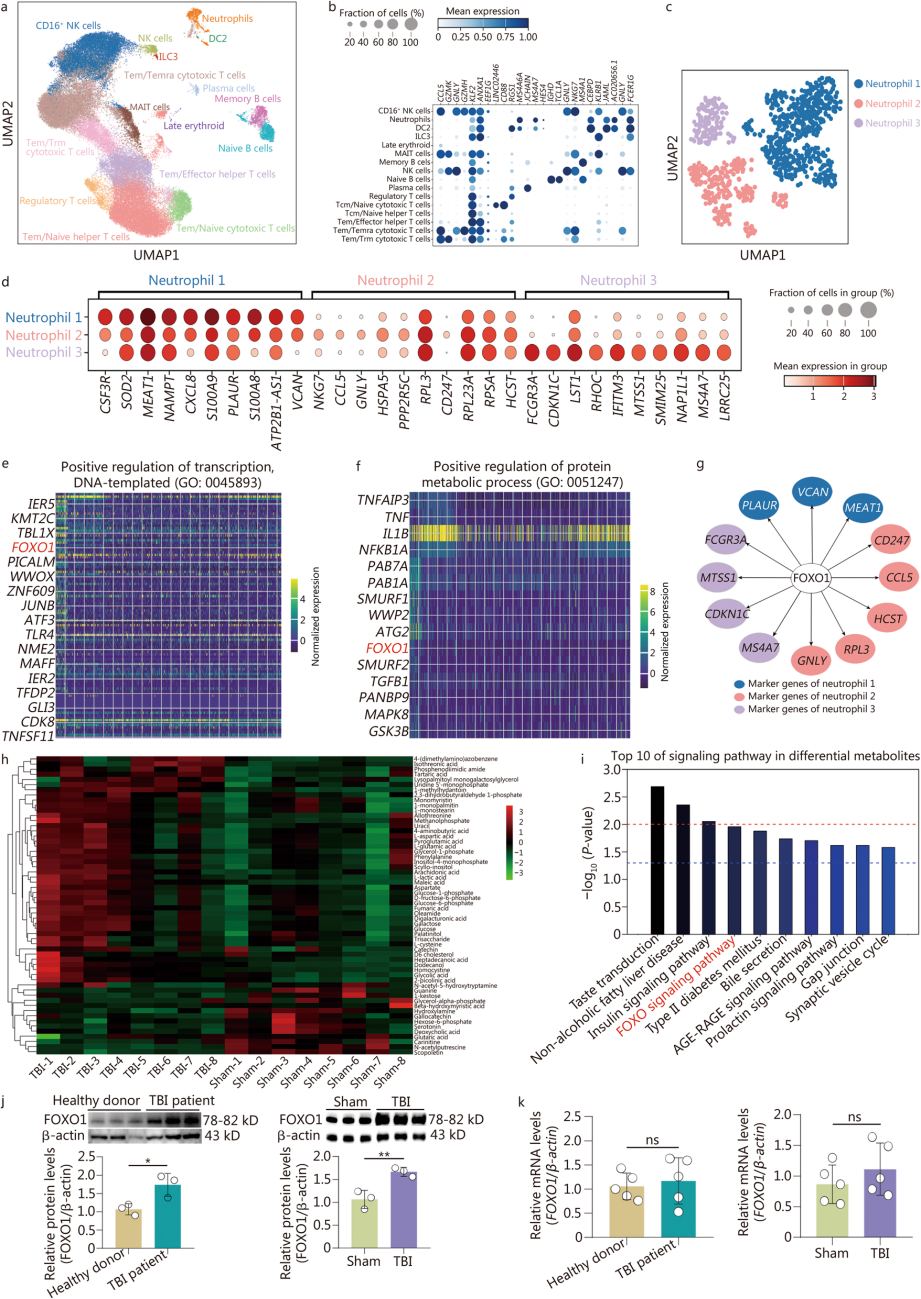
Multilevel omics integration reveals high expression of FOXO1 in the acute stage following TBI. **a** UMAP embedding of the entire dataset colored by orthogonally generated clusters labeled by manual cell type annotation, which were the scRNA-seq data analysis for peripheral blood samples from healthy donors and TBI patients (*n* = 3 in each group). **b** Dot plot showing the top 10 changed genes in every cell cluster of the human scRNA-seq.** c** UMAP plot depicting the subpopulation of human neutrophils by scRNA-seq, with each cell color-coded for clusters. **d** Dot plot showing the expression of the top 10 changed genes in each human neutrophil cluster. **e** GO analysis depicting the detailed gene of positive regulation of transcription, DNA-templated. **f** GO analysis depicting the detailed gene of positive regulation of protein metabolic process. **g** Representative regulatory network of target genes by FOXO1 in each neutrophil cluster. The same color represents the gene from the same cluster. **h** Heatmap showing the differential metabolites in neutrophils between the sham and TBI groups in mice. Higher expression levels are indicated by red and lower by green. **i** Bubble chart showing the pathway activities through the differential metabolites of neutrophils from mice (sham group vs. TBI group). Each group had 7 repeated samples of neutrophils per group. **j** Western blotting showing the expression of FOXO1 in neutrophils from humans or mice. Human: TBI patients vs. healthy donors; mice: TBI group vs. sham group, *n* = 3 for each group. **k** The relative mRNA expressions of FOXO1 in neutrophils from human or mice detected by qRT-PCR. Human: TBI patients vs. healthy donors; mice: TBI group vs. sham group, *n* = 5 for each group. Data are represented as the mean ± SEM. ^*^P < 0.05, ^**^P < 0.01, ns non-significant. FOXO1 forkhead box protein O1, TBI traumatic brain injury, UMAP uniform manifold approximation and projection, GO Gene Ontology, qRT‑PCR quantitative real‑time PCR, MAIT mucosal-associated invariant T, NK natural killer cell, DC dendritic cellsDC, ILC innate lymphoid cell, CSF3R granulocyte colony-stimulating factor receptor, SOD2 superoxide dismutase [Mn], mitochondrial, MEAT1 Terminal uridylyltransferase 7, NAMPT Nicotinamide Phosphoribosyltransferase, CXCL8 C-X-C Motif Chemokine Ligand 8, S100A9 S100 calcium binding protein A9, PLAUR plasminogen activator, urokinase receptor, S100A8 S100 calcium binding protein A8, ATP2B1-AS1 ATP2B1 antisense RNA 1, NKG7 natural killer cell granule protein 7, CCL5 C–C motif chemokine ligand 5, GNLY granulysin, HSPA5 heat shock protein family A (Hsp70) member 5, PPP2R5C protein phosphatase 2 regulatory subunit B'gamma, RPL3 ribosomal protein L3, CD247 CD247 molecule, RPL23A, ribosomal protein L23a, PSA ribosomal protein sa, HCST hematopoietic cell signal transducer, FCGR3A A fc gamma receptor iiia, CDKN1C cyclin dependent kinase inhibitor 1c, LST1 leukocyte specific transcript 1, RHOC ras homolog family member, IFITM3 interferon induced transmembrane protein 3, MTSS1 MTSS I-BAR domain containing 1, SMIM25 plaque enriched lncRNA in atherosclerotic and inflammatory bowel macrophage regulation, NAP1L1 nucleosome assembly protein 1 like 1, MS4A7 membrane spanning 4-domains a7, LRRC25 LRRC25 leucine rich repeat containing 25, IER5 immediate early response 5, KMT2C lysine methyltransferase 2c, TBL1X transducin beta like 1 x-linked, PICALM phosphatidylinositol binding clathrin assembly protein, WWOX ww domain containing oxidoreductase, ZNF609 zinc finger protein 609, JUNB JunB proto-oncogene, AP-1 transcription factor subunit, ATF3 activating transcription factor 3, TLR4 toll like receptor 4, NME2 nme/nm23 nucleoside diphosphate kinase 2, MAFF maf bzip transcription factor f, IER2 immediate early response 2, TFDP2 transcription factor dp-2, GLI3 gli family zinc finger 3, CDK8 cyclin dependent kinase 8, TNFSF11 TNF superfamily member 11, TNFAIP3 TNF α induced protein 3, TNF tumor necrosis factor, IL1B interleukin 1 β, NFKB1A nuclear factor kappa b subunit 1, SMURF1 smad specific e3 ubiquitin protein ligase 1, WWP2 WW domain containing E3 ubiquitin protein ligase 2, ATG2 autophagy related 2, SMURF2 smad specific e3 ubiquitin protein ligase 2, TGFB1 transforming growth factor β 1, PANBP9, MAPK8 mitogen-activated protein kinase 8, GSK3B glycogen synthase kinase 3 β. RAB7A member RAS oncogene family, RAB1A member RAS oncogene family, RAN binding protein 9

Because of the important role of neutrophils in TBI, we performed scRNA-seq on neutrophils from the peripheral blood of healthy donors and TBI patients within 48 h post-TBI (Additional file 1: Fig. S2a, b). After initial quality control filters, we used Harmony samples to align cell types from healthy donors and TBI patients and identified 14 clusters, including T cells, B cells, neutrophils, NK cells, dendritic cells (DCs), plasma cells and late erythroid (Fig. 1a). The gene expression levels of the clusters are shown in Fig. 1b. Focusing on neutrophils, we finally obtained approximately 2000 cells and identified 3 different subtypes of neutrophils (Fig. 1c), and their top 10 changed genes for further analysis (Fig. 1d). However, there were no special populations identified in these circulating neutrophils from TBI patients compared with those from healthy donors (Additional file 1: Fig. S2c). According to the prediction from the ChEA3 (https://maayanlab.cloud/chea3/) website, we found that most of the top 10 genes of each neutrophil cluster were the target genes of the transcription factor FOXO1. Moreover, based on the GO terms, we found that FOXO1 was the only molecule indicated to regulate both the transcription, DNA-templated (Fig. 1e), and protein metabolic process (Fig. 1f) of neutrophils from TBI patients in the acute stage. These data suggest that the FOXO1-associated gene profile was a significant characteristic of neutrophils from TBI patients (Fig. 1g). Consistent with this finding, metabolomics of murine neutrophils isolated from bone marrow after TBI showed that 58 significantly changed metabolites between neutrophils from the sham group and TBI group, according to the Kyoto Encyclopedia of Genes and Genomes pathway analysis (Fig. 1h, Additional file 1: Fig. S2d, e), the FOXO signaling pathway was significantly upregulated and activated (Fig. 1i). To confirm this hypothesis, we detected high protein rather than mRNA expression of FOXO1 in neutrophils from TBI patients and TBI mouse models, but not from healthy donors or sham group (Fig. 1j, k). This indicates that high induction of FOXO1 in neutrophils after TBI occurred at the posttranscriptional level rather than the transcriptional level, which may also explain why scRNA-seq did not identify high expression of FOXO1 mRNA in neutrophils from TBI patients. To some extent, FOXO1 protein levels in neutrophils were positively related to the injury degree of TBI in mice (Additional file 1: Fig. S2f). These data demonstrate that the presence of FOXO1^high^ neutrophils is a significant feature of neutrophils in the acute phase post-TBI and high expression of FOXO1 may orchestrate its downstream target genes to form a signaling network to regulate neutrophils in the progression of TBI.

### Interfering with FOXO1 in neutrophils alleviates neurological deficits and reduces neuronal damage in the acute stage of TBI

**Fig. 2:**
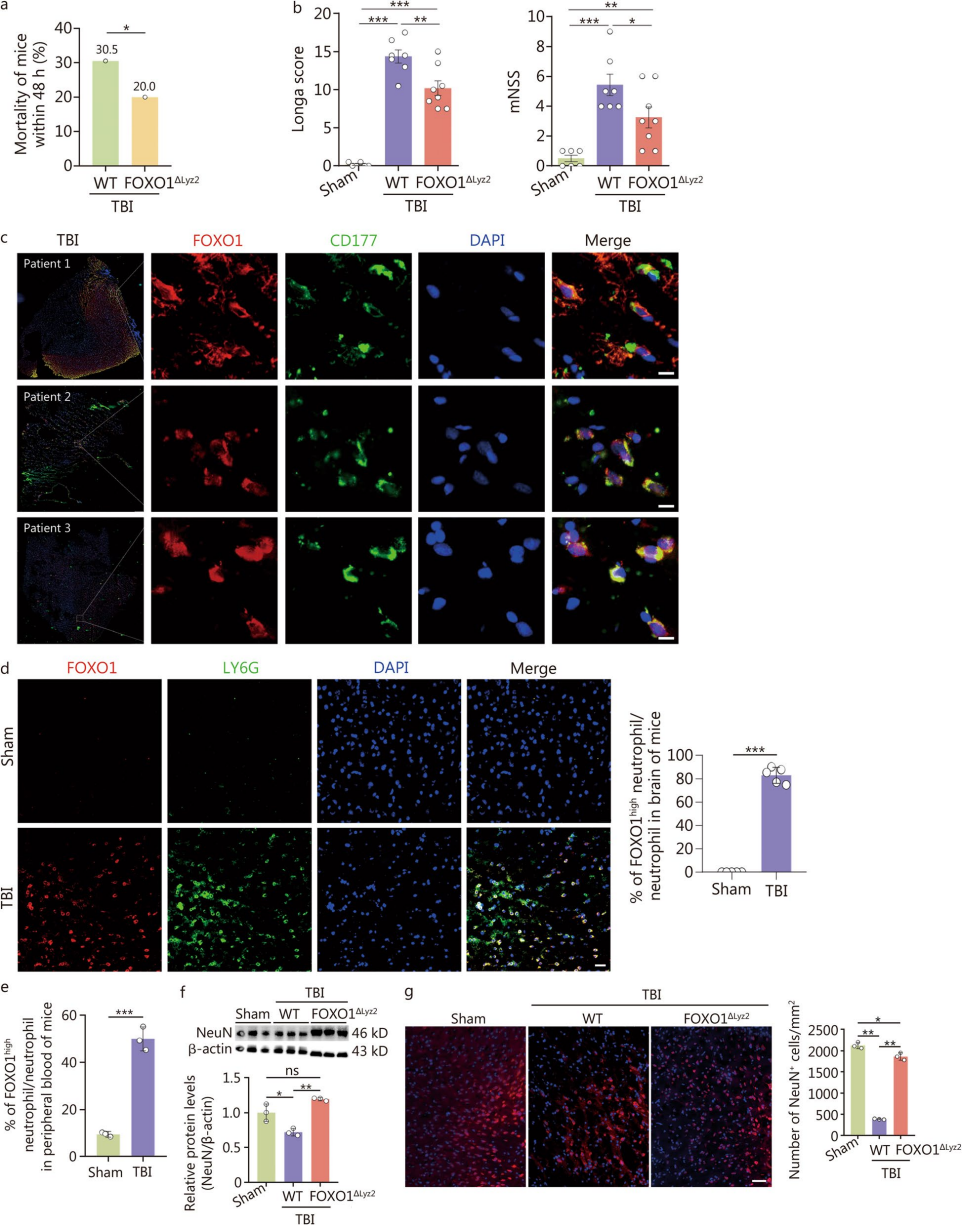
High expression of FOXO1 in neutrophils aggravates neuronal damage in the acute stage of TBI. **a** Mortality of mice within 48 h after from TBI (*n* = 50). **b** Behavioural recovery of mice was assessed using the Longa score and mNSS at 48 h post-TBI (sham group, *n* = 6; WT TBI mice, *n* = 7; FOXO1^△Lyz2^ TBI mice, *n* = 8). **c** Immunostaining of the neutrophil marker CD177 (green), FOXO1 (red), and nuclei (DAPI, blue) in brain tissue from 3 TBI patients. Scale bar = 5 μm. **d** Immunostaining of the neutrophil markers LY6G (green), FOXO1 (red), and nuclei (DAPI, blue) in brain tissue from the TBI mouse model (*n* = 5). Scale bar = 10 μm. **e** Flow cytometry analysis of the percentage of FOXO1^high^ neutrophils among leukocytes in peripheral blood of sham group and TBI mice. The experiment was repeated three times and *n* = 8 mice per group each time. **f** Western blotting showing the expression of NeuN in brain injury areas of mice. **g** Immunostaining of the neuron marker NeuN (red) and nuclei (DAPI, blue) in brain tissue from mice. Scale bar = 50 μm. In** f** and **g**, *n* = 6 per group for assay while here showed 3 biologically independent samples to represent the average status. Data are represented as the mean ± SEM. ^*^P < 0.05, ^**^P < 0.01, ^***^P < 0.001, ns non-significant. FOXO1 forkhead box protein O1, TBI traumatic brain injury, WT wild-type, mNSS modified neurological severity score, DAPI 4,6-diamidino-2-phenylindole dihydrochloride, NeuN neuronal nuclear antigen

To detect the role of FOXO1^high^ neutrophils in the acute stage of TBI, we compared the behavioral outcomes between FOXO1 conditional myeloid cell-specific knockout mice (FOXO1^△Lyz2^ mice) and their WT littermates at the time of TBI. Within 48 h post-TBI, FOXO1^△Lyz2^ mice exhibited decreased mortality and enhanced behavioral recovery, including motor coordination and balance, as well as reduced neurological deficit scores than WT littermates (Fig. 2a, b). These beneficial effects of FOXO1^△Lyz2^ mice were not observed at 24 h after TBI (Additional file 1: Fig. S3a), indicating that FOXO1 regulated neutrophils in a time-dependent manner, which requires further investigation. Moreover, we determined the heterogeneous expression of FOXO1 in neutrophils and its associated regulatory effects on neutrophil functions post-TBI. The infiltration of FOXO1^high^ neutrophils in the injured brain tissues of patients or mice was detected within or at 48 h after TBI. Immunofluorescence analysis showed that accumulated FOXO1^high^ neutrophils specifically in the traumatic brain area of TBI patients (Fig. 2c). In line with this, our findings in the mouse model of TBI revealed that approximately 80% of infiltrated neutrophils in injured brain tissue exhibited high levels of FOXO1 expression at 48 h after TBI (Fig. 2d). The presence of FOXO1^high^ neutrophils in the peripheral blood was also demonstrated via flow cytometry (Fig. 2e, Additional file 1: Fig. S3b), and the number of FOXO1^high^ neutrophils increased with the severity of injury (Additional file 1: Fig. S3c). Administration of FOXO1 inhibitor AS1842856 mimicked the protective effects on neurological deficits as those in FOXO1^△Lyz2^ mice, providing insights into potential pharmacological or genetic approaches to target FOXO1 could pave the way for future therapeutic development (Additional file 1: Fig. S3d, e).

It is well known that trauma-induced brain injury results in tissue damage and neuronal loss [11]. The results of our study demonstrated that FOXO1^△Lyz2^ mice exhibited a significant reduction in TBI-induced neuron loss in vivo (Fig. 2f, g). To strengthen the specificity of FOXO1-mediated effects, we have made the comparative analysis using the APPswe/PS1dE9 (APP/PS1) mouse model of Alzheimer’s disease. The results showed that there were few FOXO1^high^-neutrophils in the brain tissues of the mouse model for Alzheimer’s disease (Additional file 1: Fig. S3f). Taken together, these findings indicate that FOXO1^high^ neutrophils aggravated neurological deficits and brain damage in the acute stage of TBI.

### FOXO1 enhances the anti-apoptosis abilities with IL-6 production of neutrophils via “FOXO1-VCAN” signalling

**Fig. 3:**
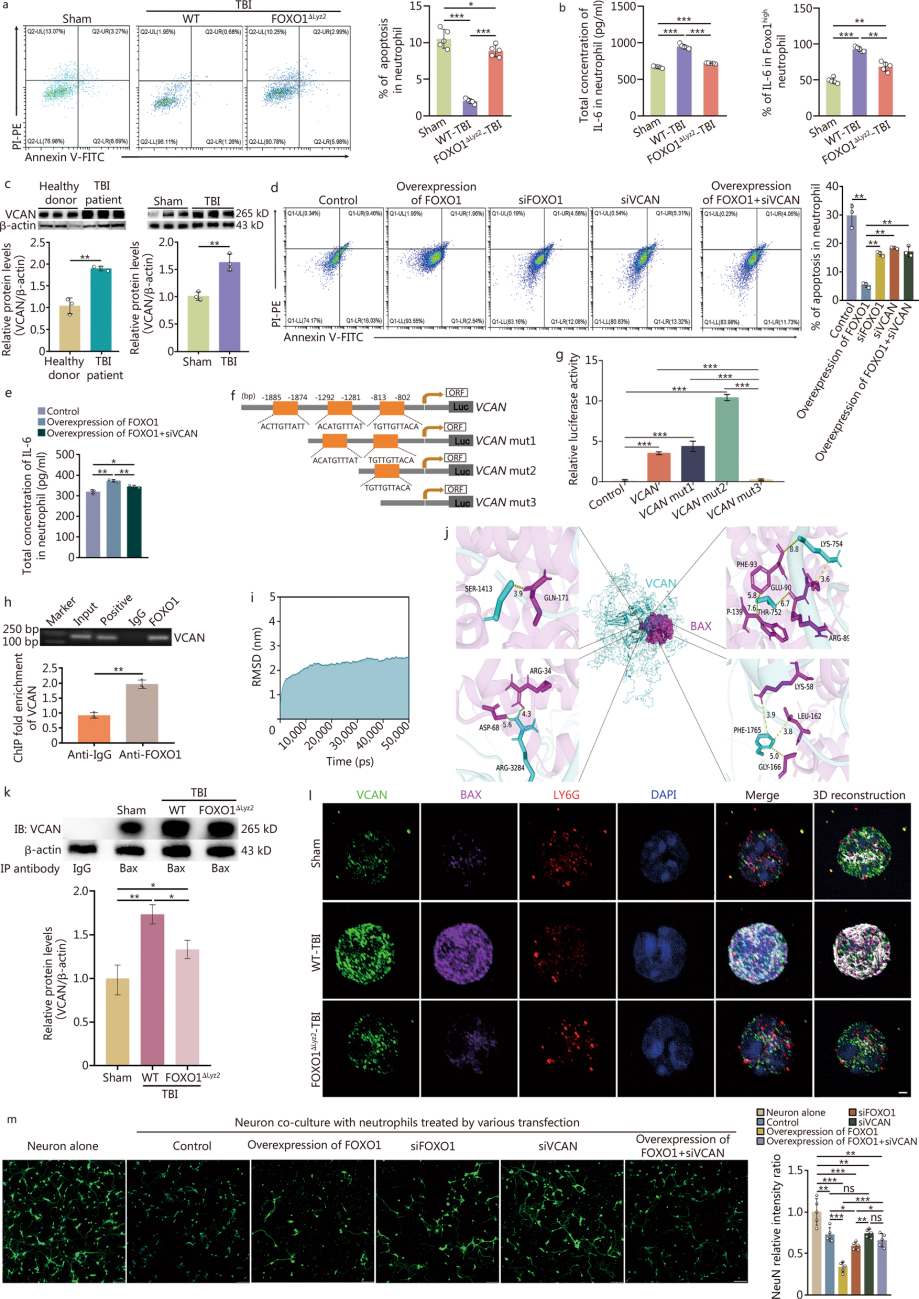
FOXO1 enhances the anti-apoptosis and IL-6 release abilities of neutrophils. **a** Flow cytometry showing the percentage of apoptosis in neutrophil infiltrate to brain injury in TBI mice (*n* = 5). **b** ELISA analysis (left) and flow cytometry (right) of IL-6 levels in neutrophils of mice collected after TBI (*n* = 5). **c** Western blotting showing the expression of VCAN in neutrophils in both TBI patients and TBI mouse models. Three biologically independent samples of each group represented the average status here. **d** Flow cytometry showing the percentage of apoptosis in neutrophils transfected with different over/si-expression plasmids. Three biologically independent samples to represent the average status. **e** ELISA analysis of IL-6 in neutrophils of mice. The experiment was repeated three times. **f** Putative FOXO1 binding sequence of the mouse *VCAN* promoter gene. *VCAN* mut 1: deletion of one FOXO1 binding site at − 1874 to − 1885 bp; *VCAN* mut 2: deletion of two FOXO1 binding sites at − 1874 to − 1885 bp and − 1281 to − 1292 bp; *VCAN* mut 3: deletion of three FOXO1 binding sites at − 1874 to − 1885 bp, − 1281 to − 1292 bp and − 802 to − 813 bp. **g** Luciferase activity of FOXO1 co-transfected with mutated reporters under specific conditions. All transfected cells were treated under the indicated conditions for 6 h and lysed for dual-luciferase measurements. **h** ChIP of the FOXO1 binding sequence from the murine VCAN promoter gene. After treating neutrophils with the indicated conditions for 6 h, the total chromatin was collected and amplified as input (positive control). Antibodies against FOXO1 were used to pull down the binding segments, of which IgG was introduced as a negative control. **i** The RMSD showed by the backbone atoms of the VCAN-BAX system during the molecular docking. **j** Pymol MOE software was used to predict the binding between VCAN and BAX. **k** Co-IP of VCAN and BAX in neutrophils from bone marrow in a mouse model. β-actin was detected in the supernatant after IP. **l** Immunostaining showing the expression, location, and binding between BAX and VCAN in neutrophils from bone marrow in the mouse model of TBI. Scale bar = 1 μm. **m** Immunostaining showing the expression of the neuron marker NeuN (green) and nuclei (DAPI, blue) in neurons co-cultured with neutrophils treated as described above. Scale bar = 50 μm. The experiment was repeated 5 times. Data are represented as the mean ± SEM. ^*^P < 0.05, ^**^P < 0.01, ^***^P < 0.001, ns non-significant. FOXO1 forkhead box protein O1, TBI traumatic brain injury, ELISA enzyme‐linked immunosorbent assay, ORF open reading frame, mut mutant, ChIP chromatin immunoprecipitation, VCAN Versican, RMSD root mean square deviation, BAX BCL-2-associated X protein, DAPI 4,6-diamidino-2-phenylindole dihydrochloride, Co-IP co-immunoprecipitation

To investigate the role of FOXO1 in regulating neutrophils after TBI, we further analyzed the scRNA-seq data from TBI patients using gene set enrichment analysis [30], an analytical method focusing on gene sets that share common biological functions and regulation. Based on gene rank, we obtained the top 10 biological pathways (Additional file 1: Fig. S4a). Most notably, apoptosis regulated by the apoptosis regulator BCL-2 family was significantly enriched (Additional file 1: Fig. S4b). The flow cytometry analysis demonstrated that neutrophils derived from TBI mice showed better anti-apoptotic ability, which was attenuated by *FOXO1* conditional knockout (Fig. 3a), indicating that high expression of FOXO1 contributes to extending the lifespan of neutrophils in TBI. In addition to exerting the anti-apoptotic effect, FOXO1 also significantly modulated the capacity of neutrophils to release the pro-inflammatory cytokine IL-6 rather than TNF-α and IL-1β (Fig. 3b, Additional file 1: Fig. S4c–e). These data suggest that FOXO1 prolongs the lifespan of neutrophils by increasing its pro-inflammatory effect after TBI.

In an effort to explore the underlying molecular mechanism, we verified the target genes of FOXO1 in neutrophils indicated by scRNA-seq as described in Fig. 1g. VCAN was specifically highly expressed in neutrophils from both TBI patients and the mouse model of TBI (Fig. 3c; Additional file 1: Fig. S4f, g). Additionally, further scRNA-seq analysis found that VCAN exhibited high expression exclusively in circulating neutrophils of TBI patients while remaining undetectable in other circulating immune cells (Additional file 1: Fig. S4h). As expected, interfering with VCAN expression in FOXO1^high^ neutrophils blocked the FOXO1-mediated anti-apoptotic effect and IL-6 production in neutrophils (Fig. 3d, e). Combined with bioinformatics analysis, luciferase assay, and ChIP experiment, we identified that VCAN is directly and specifically bound to FOXO1 at the site of − 802 to − 813 bp of its promoter region (Fig. 3f–h). Taken together, these data confirmed that *VCAN* is a target gene involved in neutrophil FOXO1 function. VCAN is an important subgroup of chondroitin sulfate proteoglycans, usually secreted extracellularly serving as extracellular matrix molecules [31]. However, in the present study, we found that FOXO1 upregulated VCAN in the cytoplasm and enhanced its interaction with the proapoptotic protein apoptosis regulator BAX, thereby inhibiting BAX translocation to the mitochondria to form the proapoptotic BAX-BCL-2 complex (Fig. 3i–l, Additional file 1: Fig. S4i), demonstrating that FOXO1 triggers the “FOXO1-VCAN” signaling to reprogram neutrophils and enhance their anti-apoptosis effect as well as IL-6 production. In the neutrophil and neuron co-culture system in vitro, we showed that FOXO1^high^ neutrophil-induced neuron damage could be blocked by siVCAN (Fig. 3m), providing further evidence for the involvement of this mechanism in promoting acute-phase neuronal damage following TBI.

Generally, FOXO1 is considered to regulate cell activity by affecting metabolism. Here, the application of metabolomics and enzyme-linked immunosorbent assay showed significant changes in related metabolites and key enzymes involved in glucose metabolism within neutrophils of the TBI mouse model, indicating an increase in the tricarboxylic acid cycle (Additional file 1: Fig. S5a, b). We also found that FOXO1^high^ neutrophils exhibit a significant increase in ATP production and oxygen consumption, which is mediated by FOXO1 (Additional file 1: Fig. S5c, d), indicating the enhancement of aerobic oxidation. Since tricarboxylic acid cycle/aerobic oxidation is the key metabolic pathway of mitochondria, we next assayed the changes of mitochondria in FOXO1^high^ neutrophils. The results showed that FOXO1 mediated the increase of mitochondria number and mitochondrial membrane potential while decreasing the expression of optic atrophy 1 (OPA1) (Additional file 1: Fig. S5e–g). OPA1 has been reported to be important for maintaining glycolytic ATP production in neutrophils [32]. Therefore, the low levels of OPA1 observed in FOXO1^high^ neutrophils indicated these cells mainly depend on other patterns of energy metabolism, such as aerobic oxidation rather than glycolysis. Taken together, these data indicate that FOXO1 promotes a shift from glycolysis to aerobic oxidation in FOXO1^high^ neutrophils and provide evidence for a connection between their metabolic reprogramming and altered function post-TBI.

### Infiltration of FOXO1^high^ neutrophils in the brain during the chronic phase of TBI is closely associated with the incidence of depression

**Fig. 4:**
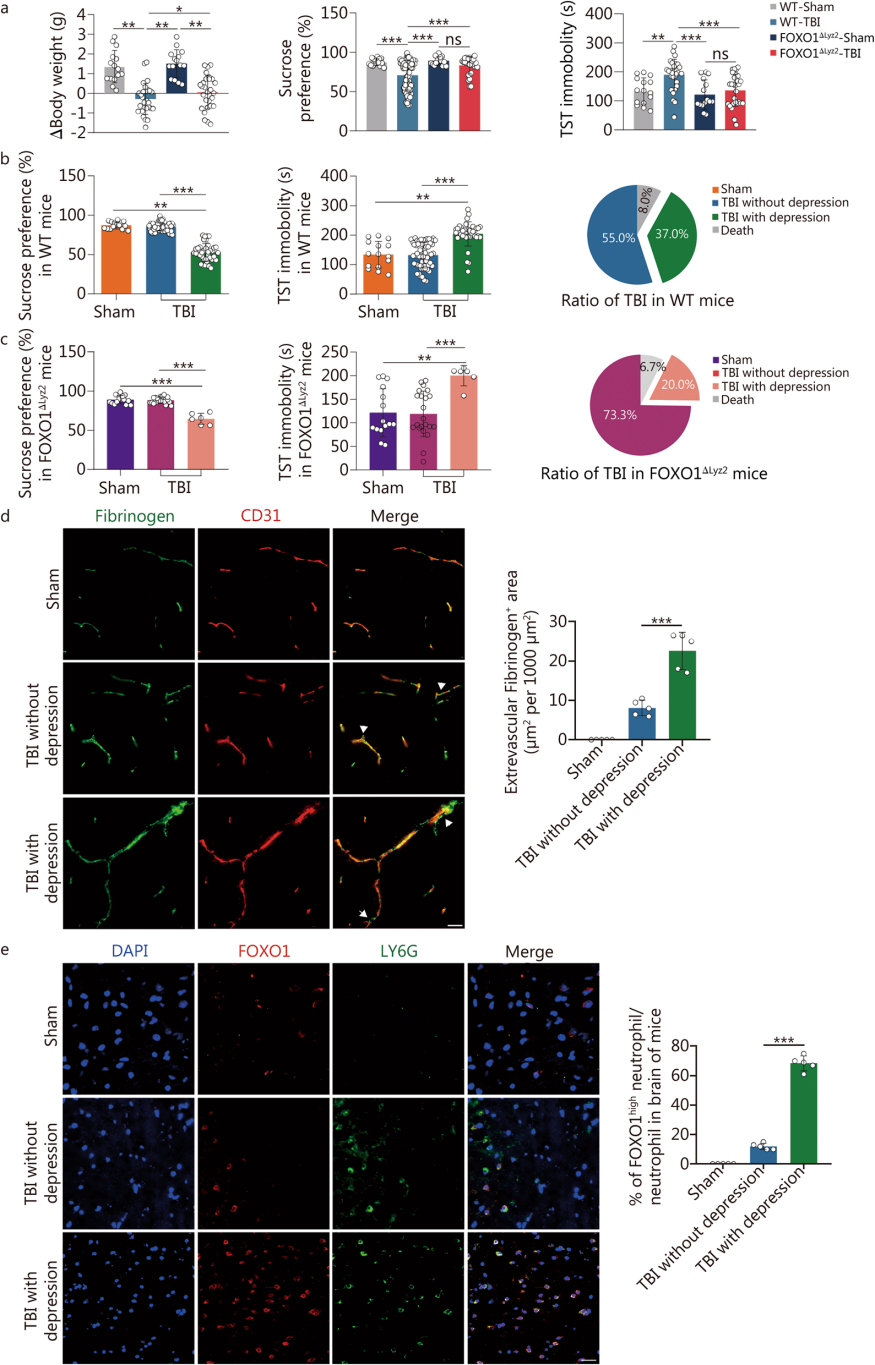
High expression of FOXO1 in neutrophils reduces the incidence of depression after TBI. FOXO1^△Lyz2^ mice and their WT littermate were randomly divided into a sham group and a TBI group. **a** Body weight and behavioral tests, including the sucrose preference and TST in mice, *n* = 30 in the sham group, *n* = 100 in WT TBI mice, *n* = 30 in FOXO1^△Lyz2^ sham mice, and *n* = 30 in FOXO1^△Lyz2^ TBI mice. **b** The behavioral tests of sucrose preference and TST were assayed in WT TBI mice for the identification of depression after TBI. Sham group, *n* = 15; TBI with depression group, *n* = 37; TBI without depression group, *n* = 55. **c** The behavioral tests of sucrose preference and TST were assayed in FOXO1^△Lyz2^ TBI mice for the identification of depression after TBI. Sham group, *n* = 15; TBI with depression group, *n* = 6; TBI without depression group, *n* = 22. **d** Immunostaining of fibrinogen (green) and CD31 (red) in the brain tissue of TBI mice (scale bar = 20 μm). **e** Immunostaining of FOXO1 (red), LY6G (green), and nuclei (DAPI, blue) in brain injury tissue of the TBI mouse model (scale bar = 20 μm). In **d** and **e**, in each group, *n* = 5 for data collection. Data are represented as the mean ± SEM. ^*^P < 0.05, ^**^P < 0.01, ^***^P < 0.001, ns non-significant. FOXO1 forkhead box protein O1, TBI traumatic brain injury, WT wild-type, TST tail suspension test, DAPI 4,6-diamidino-2-phenylindole dihydrochloride

Depression is one of the most common complications after TBI with high incidence in the first year and persists at a high rate even up to 50 years after the injury [6, 7]. We investigated whether TBI caused depressive-like behavior 1 month after moderate TBI. In the TBI mouse model, mice with TBI-induced depression were identified based on indexes including exaggerated weight loss, less sucrose intake, and increased total immobility time of tail suspension test. Firstly, we confirmed there was no significant difference in the anesthetic recovery time after TBI between WT and FOXO1^△Lyz2^ mice, excluding the effect of FOXO1 on anesthesia-related neurological damage and psychological problems (Additional file 1: Fig. S6a). Next, we observed that FOXO1^△Lyz2^ mice had a markedly decreased incidence of depression 1 month after TBI, from 37.0 to 20.0% (Fig. 4a-c). Additionally, the extravascular accumulation of fibrinogen in the injured brain cortical tissue was significantly higher in depressed mice compared to non-depressed mice after TBI (Fig. 4d), suggesting persistent impairment of the BBB in mice with TBI and depression. Even in the chronic stage of TBI, disruption of BBB promotes the infiltration of circulating immune cells into the brain. Therefore, we assayed the infiltration of neutrophils in these mice with or without TBI-induced depression after TBI. To our surprise, immunostaining results revealed a substantial accumulation of FOXO1^high^ neutrophils in the injured brain areas of mice with TBI-induced depression (Fig. 4e). It is further confirmed that the presence and infiltration of FOXO1^high^ neutrophils in the chronic stage of TBI is involved in TBI-induced depression. Indeed, we consistently and dynamically detected the disruption of BBB and infiltration of FOXO1^high^ neutrophils in certain mice (Additional file 1: Fig. S6b, c). Moreover, in vitro experiment confirmed that FOXO1^high^ neutrophils were implicated in the disruption of BBB by reducing the expression of zonula occludens-1 (ZO-1), a tight junction-actin cytoskeleton connecting protein in brain endothelial cells (Additional file 1: Fig. S6d). Furthermore, by using FOXO1 inhibitor AS1842856 to mimic the effect of FOXO1^△Lyz2^ mice, we found that AS1842856 also attenuated some neurological deficits including sucrose preference and tail suspension test in TBI-induced depressive mice, although not as effective as conditional *FOXO1* knockout on neutrophils (Additional file 1: Fig. S6e, f). These data collectively suggest that FOXO1^high^ neutrophils also play an important role in contributing to chronic or long-term brain damage post-TBI.

### FOXO1^high^ neutrophils significantly reduce the expression of MBP on oligodendrocytes

**Fig. 5:**
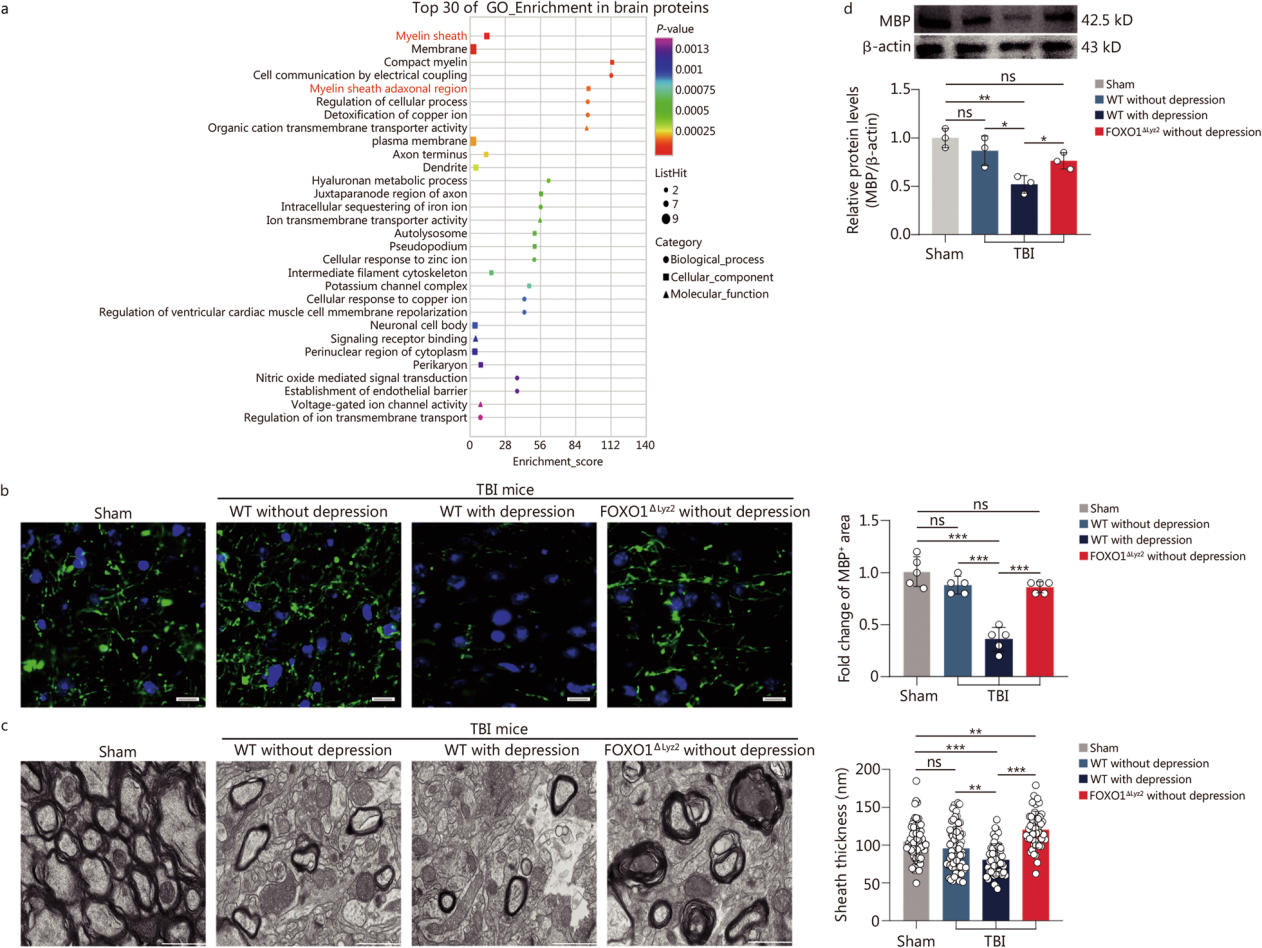
High expression of FOXO1 in neutrophils increases myelin damage. **a** Proteomic analysis of brain tissues from mice. The top 30 proteins are enriched terms by GO analysis (group of TBI with depression vs. group of TBI without depression in WT mice). **b** Immunostaining of MBP (green) and nuclei (DAPI, blue) in the brain tissue of different groups (*n* = 5). Scale bar = 50 μm. **c** Electron microscopy image of corpus callosum sections and quantification of myelinated axon number and g-ratio (*n* = 6). Scale bar = 1 μm. **d** Western blotting showing the expression of MBP in neutrophils in the TBI mouse model (*n* = 3). In each group, three biologically independent samples were represented. Data are represented as the mean ± SEM. ^*^P < 0.05, ^**^P < 0.01, ^***^P < 0.001, ns non-significant. FOXO1 forkhead box protein O1, GO Gene Ontology, TBI traumatic brain injury, MBP myelin basic protein, DAPI 4,6-diamidino-2-phenylindole dihydrochloride

In order to elucidate the underlying molecular mechanisms by which FOXO1 regulates neutrophils in depression induced by TBI, we employed proteomics methods to analyze and compare the changes of protein in injured brain tissues of mice with or without TBI-induced depression. The GO analysis indicated that the most significant change in cellular function was in the myelin sheath, including compact myelin and the myelin sheath adaxonal region (Fig. 5a). Notably, the levels of MBP, key structural elements of the myelin sheath [33], were significantly decreased in mice with TBI-induced depression. Several studies have demonstrated that myelin abnormalities are closely related to the occurrence and progression of depression in both patients and mice [34–36]. The immunostaining and transmission electron microscopy images showed a decrease in the number and thickness of myelin in depressed mice after TBI compared to non-depressed mice. However, these pathological changes were alleviated in *FOXO1* conditional knockout mice (Fig. 5b, c). The Western blotting results also confirmed that the expression of MBP was significantly reduced in the brain tissues of depressed mice after TBI compared with that of mice without depression at this time point (Fig. 5d). These data indicate that the decrease of MBP expression is involved in the promoting role of FOXO1^high^ neutrophils in TBI-induced depression.

### FOXO1-disrupted iron homeostasis between neutrophils and oligodendrocytes results in a decrease in MBP in oligodendrocytes

**Fig. 6:**
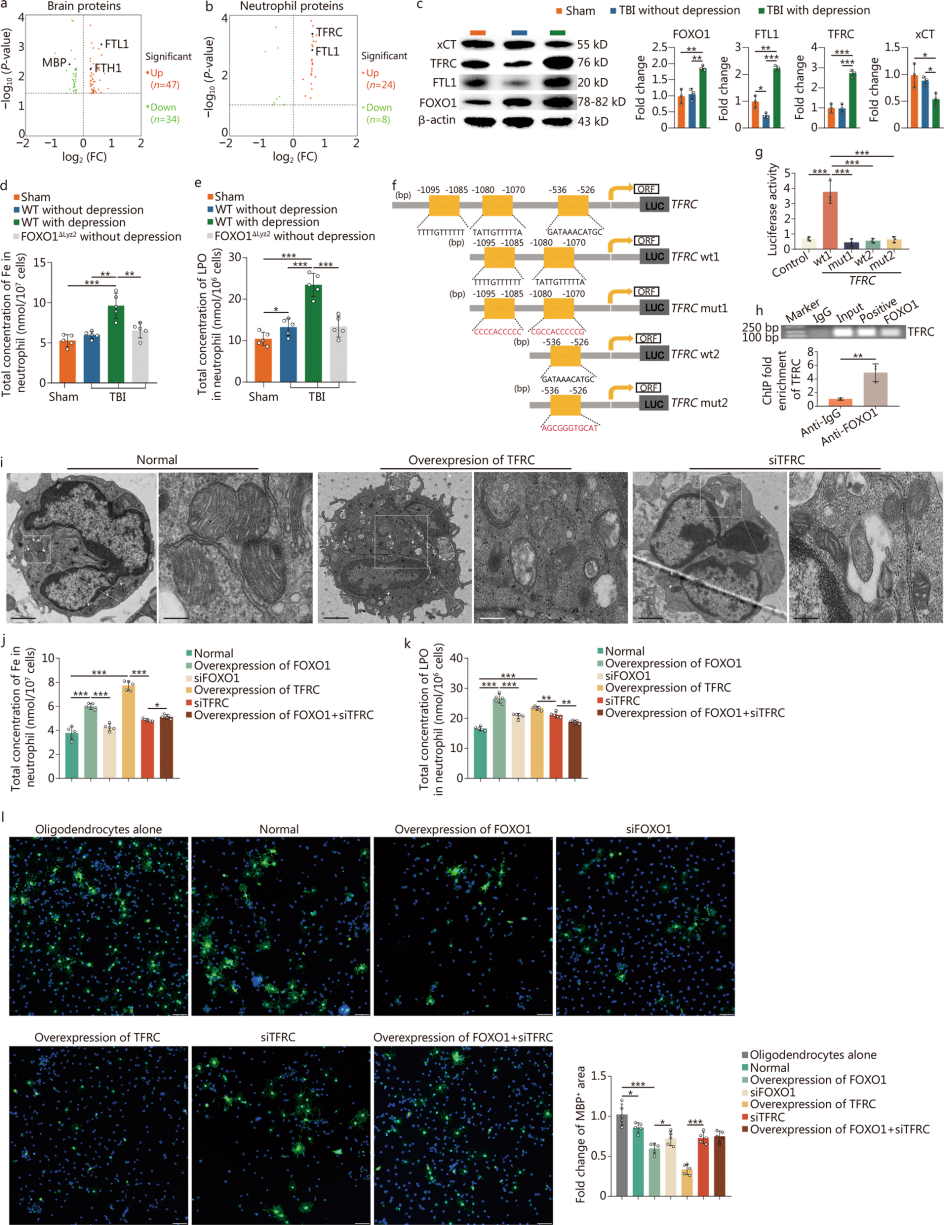
FOXO1 disrupts iron homeostasis in neutrophils and oligodendrocytes. **a** Volcano plot showing variation in protein expression in brain-insured tissues between the group of TBI with depression and the group of TBI without depression in WT mice. The fold change (FC) log (base 2) is on the X-axis, and the negative false log discovery rate (*P-*value) (base 10) is on the Y-axis. Higher expression levels are indicated by red and lower by green. **b** Volcano plot showing variation in protein expression of neutrophils between the TBI-induced depression group and the insusceptible group. The FC log (base 2) is on the X-axis, and the negative false log discovery rate (*P*-value) (base 10) is on the Y-axis. Higher expression levels are indicated by red and lower by green. **c** Western blotting showing the expression of FOXO1, FTL11, TFRC, and xCT in neutrophils from the TBI mice with and without depression (*n* = 6). The sham group served as the control. In each group, three biologically independent samples were represented here to show the average status. **d** Intracellular iron colorimetric assay showing the concentration of total Fe in neutrophils from bone marrow of the TBI mice with and without depression (*n* = 5). The sham group served as the control. **e** LPO assay showing the concentration of LPO in neutrophils from the bone marrow of the TBI mice with and without depression (*n* = 5). The sham group served as the control. **f** Putative FOXO1 binding sequence of the mouse *TFRC* promoter gene. *TFRC* wt1: the reporter plasmid containing the two FOXO1 binding sites at − 1070 to − 1080 bp and − 1085 to − 1095 bp; *TFRC* mut1: the reporter plasmid containing the two mutated FOXO1 binding sites at − 1070 to − 1080 bp and − 1085 to − 1095 bp; *TFRC* wt2: the reporter plasmid containing the FOXO1 binding site at − 526 to − 536 bp; *TFRC* mut2: the reporter plasmid containing the mutated FOXO1 binding site at − 526 to − 536 bp. **g** Luciferase activity of FOXO1 co-transfected with mutated reporters under specific conditions. All transfected cells were treated under the indicated conditions for 6 h and lysed for dual-luciferase measurements. **h** ChIP of the FOXO1 binding sequence from the murine TFRC promoter gene. After treating neutrophils with the indicated conditions for 6 h, the total chromatin was collected and amplified as input (positive control). Antibodies against FOXO1 were used to pull down the binding segments, of which IgG was introduced as a negative control. **i** Electron microscopy image of the form of mitochondria in neutrophils. **j** Intracellular iron colorimetric assay showing the concentration of total Fe in neutrophils. **k** LPO assay showing the concentration of LPO in neutrophils from bone marrow. **l** Immunostaining of MBP (green) and nuclei (DAPI, blue) in oligodendrocytes (scale bar = 50 μm). Each experiment was repeated three times. Data are represented as the mean ± SEM. ^*^P < 0.05, ^**^P < 0.01, ^***^P < 0.001. FOXO1 forkhead box protein O1, TBI traumatic brain injury, wt wild-type, FTH1 ferritin heavy chain 1, FTL1 ferritin heavy chain 1, TFRC transferrin receptor, xCT system xc(−) cystine/glutamate antiporter, mut mutant, LPO lipid hydroperoxide, ChIP chromatin immunoprecipitation, MBP myelin basic protein, DAPI 4,6-diamidino-2-phenylindole dihydrochloride

By examining brain tissue proteomics and neutrophils isolated from bone marrow in mice with or without TBI-induced depression, we found evidence of disrupted iron metabolism at the “cellular process” level, potentially linked to ferroptosis. The results showed that ferroptosis-associated proteins, including ferritin heavy chain 1 (FTH1)/ferritin light chain 1 (FTL1) in brain tissues and FTL1/TFRC in neutrophils, were both increased (Fig. 6a, b; Additional file 1: Fig. S7). The expression of these proteins in TBI-induced depression mice was further demonstrated through Western blotting (Fig. 6c). By detecting the system xc(−) cystine/glutamate antiporter (xCT) level, total Fe concentration and LPO, we confirmed that ferroptosis levels were significantly increased in bone marrow neutrophils of TBI-induced depression mice, which was significantly alleviated by conditional knockout of *FOXO1* in neutrophils (Fig. 6c-e).

The mechanism of FOXO1-associated ferroptosis in neutrophils was investigated through bioinformatics analysis, luciferase assays, and ChIP experiments. The results showed that *TFRC* is a target gene of FOXO1 in TBI-induced depressed neutrophils, with direct and specific binding to FOXO1 at the sites of − 1070 to − 1095 bp of its promoter region (Fig. 6f-h). The ferroptosis of neutrophils induced by FOXO1 overexpression was inhibited in vitro through siRNA-mediated knockdown of *TFRC* expression (Fig. 6i-k), indicating that “FOXO1-TFRC” is involved in the disruption of iron homeostasis in neutrophils during the chronic stage of TBI. Furthermore, in a co-culture system of neutrophils and oligodendrocytes, we observed that these “FOXO1-TFRC” signaling-regulated neutrophils were responsible for the abnormalities in iron levels and FTH expression, which was closely associated with the decrease of MBP in oligodendrocytes (Fig. 6l). Altogether, these findings revealed that dysregulated iron metabolism mediated by FOXO1 between neutrophils and oligodendrocytes results in a decrease in MBP in oligodendrocytes, thereby contributing to the development of depression following TBI.

## Discussion

**Fig. 7:**
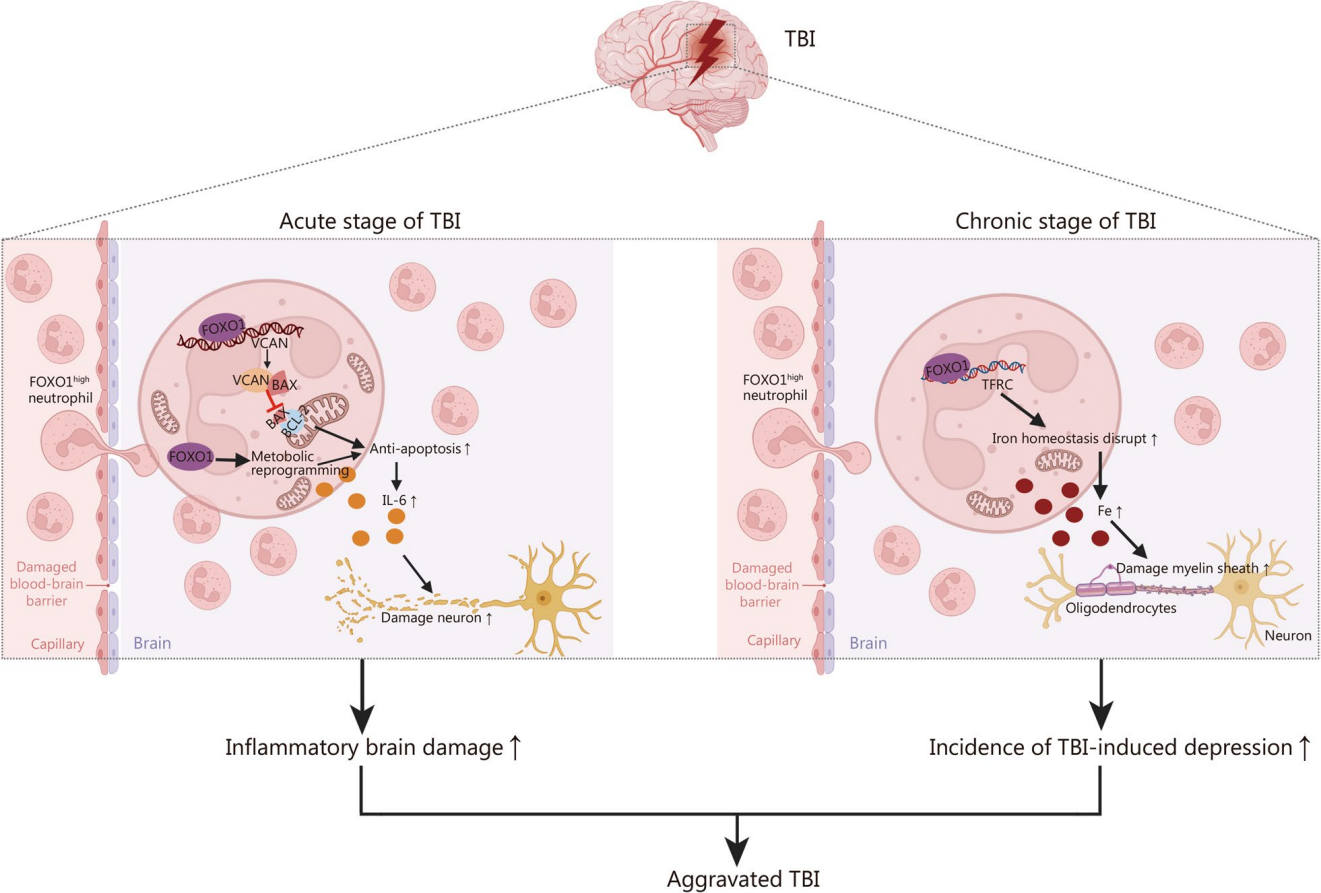
The effects and mechanism of FOXO1 high neutrophils in the acute and chronic stages of TBI. FOXO1 forkhead box protein O1, TBI traumatic brain injury, VCAN Versican, TFRC transferrin receptor, MBP myelin basic protein, BAX B-cell lymphoma-2 (BCL-2)-associated X protein, BCL-2 B-cell lymphoma-2, IL-6 interleukin-6

The recruitment of neutrophils following injury is a crucial early event in the pathogenesis of TBI. In this study, we found that neutrophils with high levels of FOXO1 expression (FOXO1^high^ neutrophils) infiltrate brain tissue and aggravate brain damage in both acute and chronic stages post-TBI. FOXO1 directly promotes brain injury during the acute phase response after trauma by upregulating the novel target gene, *VCAN*, thereby enhancing the neutrophils’ anti-apoptotic ability and increasing IL-6 production. In the chronic phase after TBI, there is a significant positive correlation between the persistence and infiltration of FOXO1^high^ neutrophils in the brain and the incidence of TBI-induced depression in mice. The pro-depressive effect of FOXO1^high^ neutrophils arises from abnormal iron metabolism mediated by FOXO1 between neutrophils and oligodendrocytes, ultimately leading to a decrease in MBP in oligodendrocytes. Collectively, FOXO1 exerts a pivotal role in the progression of both acute and chronic phases of TBI by modulating neutrophil lifespan, cytokine production, and iron metabolism via intricate signaling networks (Fig. 7).

Neutrophils have traditionally been thought to have low RNA content, exhibit a lifespan of less than 24 h in homeostatic conditions, and experience rapid aging in peripheral circulation and tissues. Accordingly, they were thought to be homogeneous, and incapable of adapting to changing conditions [37]. However, with advances in genomics technology, especially single-cell omics sequencing, the extent of cellular heterogeneity across different tissues and cell types has become increasingly apparent. Recently, numerous studies have suggested that neutrophils are functionally and phenotypically heterogeneous, mainly under pathological states. A comprehensive transcriptional landscape of neutrophil maturation, function, and fate decisions during mouse steady state and bacterial infection was reported [38]. The study conducted by Kwok et al. [39] demonstrated the presence of an early-directed neutrophil progenitor within granulocyte-monocyte progenitors, which specifically underwent expansion during emergency granulopoiesis in sepsis. Wang et al. [40] showed that there were three distinct neutrophil subsets associated with tumors, as well as basic helix-loop-helix family member e40 (BHLHE-40)-driven pro-tumor neutrophils in the pancreatic tumor microenvironment, which exhibit hyperactivated glycolysis. In this study, we combined scRNA-seq of circulating neutrophils from TBI patients with proteomics analysis of neutrophils from a TBI mouse model, and found a high expression of FOXO1 protein in neutrophils. In both patients and the TBI mouse model, these FOXO1^high^ neutrophils accounted for approximately 60% of circulating neutrophils and 80% of infiltrated neutrophils at 48 h post-TBI. Surprisingly, these FOXO1^high^ neutrophils exerted anti-apoptotic activity accompanied by an increase in IL-6 release, which could potentially induce the aggregation and activation of other immune cells, thereby amplifying the cascade release of inflammatory mediator and exacerbating brain damage. A previous study has extensively investigated the role of FOXO1 in glucose metabolism, cell behavior, and susceptibility to infection, implicating its involvement in the pathogenesis and progression of various diseases, such as cancer, liver steatosis, and atherosclerosis [14]. Although some essential modulatory effects of FOXO1 on leukocytes have been reported in metabolic diseases and bacterial infection [41, 42], its role in TBI and the regulation of neutrophils remains poorly understood. Herein, through in vitro and in vivo experiments, we first demonstrated that the detrimental effects of FOXO1^high^ neutrophils in TBI are dependent on FOXO1.

In the acute stage of TBI, we found that FOXO1 exerts an anti-apoptotic effect on neutrophils by regulating its target gene *VCAN*. VCAN is an extracellular matrix proteoglycan that interacts with cells by binding to nonintegrin and integrin receptors, and it is expressed both in the cytoplasm and on the cell surface [43]. Recent studies have reported that VCAN promotes cell proliferation and apoptosis, which are related to activation of the EGFR-PI3K-Akt pathway or downregulation of BCL-2-associated agonist of cell death expression [31, 44]. These findings suggest that the paradoxical and complex effects of VCAN on cell lifespan may stem from different underlying pathological (disease) processes. In this study, we identified that VCAN acts as a direct target of FOXO1. Upregulation of cytoplasmic VCAN enhanced its interaction with BAX, thereby inhibiting BAX mitochondrial translocation and binding to BCL-2. The “FOXO1-VCAN” signaling pathway mediates the anti-apoptosis of neutrophils post-TBI. Our results support the opinion that neutrophils possess heterogeneous immunophenotypes and dynamic functional plasticity, rather than being terminally differentiated cells with limited gene expression regulation capacity. We also observed a shift in FOXO1-dependent glucose metabolism from glycolysis to aerobic oxidation in FOXO1^high^ neutrophils. However, it is not clear whether this reprogramming is also involved in the prolonged lifespan of neutrophils, requiring further investigation.

It is widely acknowledged that neutrophils accumulate in injured tissues during the acute phase of inflammation [45]. Recent evidence has suggested their crucial role in initiating and shaping immune response during more chronic inflammatory diseases, such as cancer, autoimmune disease, and atherosclerosis [46]. Bye et al. [47] reported a continuous infiltrate of neutrophils into the lesion site and the surrounding cortex of brain tissue 4 d following TBI. Kenne et al. [48] further demonstrated that depletion of neutrophils reduced oedema formation and tissue damage 14 d after TBI in mice. These findings have raised concerns about the roles of neutrophils in the chronic stage of TBI; however, their specific functions and underlying molecular mechanisms remain largely unknown and unexplored. Our study surprisingly found a strong association between the infiltration of FOXO1^high^ neutrophils during this stage and TBI-induced depression. Given the critical role of neuroinflammation and BBB integrity in TBI pathophysiology, we consistently observed BBB disruption alongside FOXO1^high^ neutrophil infiltration after TBI in mice. In conjunction with the impairment of tight junctions in cultured brain endothelial cells caused by FOXO1^high^ neutrophil, it is suggested that the interplay between FOXO1^high^ neutrophil, neuroinflammation and BBB integrity contributes to long-term damages associated with TBI.

Traumatic depression is characterized by several cellular and molecular mechanisms including elevated IL-6 and TNF-α in plasma [49], reduced brain-derived neurotrophic factor, which may aggravate neuronal damage [50], and the reactivity of microglial 30 d after TBI is associated with the manifestation of depressive-like behavior [51]. Among them, mechanisms, dysfunction, or demyelination mediated by oligodendrocytes play a significant role in the development of depression [52–54]. Our findings demonstrate that FOXO1 disrupts the ions homeostasis in FOXO1^high^ neutrophils by regulating TFRC. In humans, TFRC encodes transferrin receptor protein 1 (UniProt-P02786), which controls intracellular iron levels. Transferrin receptor protein 1 imports extracellular iron into cells to establish a cellular iron pool crucial for ferroptosis [55]. This study revealed that *TFRC*, as a target gene of FOXO1, exhibits significant expression in neutrophils and increases FTH1, a cytosolic iron storage protein involved in multiple physiological processes including ferroptosis. Wang et al*.* [56] demonstrated that local tissue activation of FTH1 leads to neutrophil ferroptosis and aggravates inflammation in acute lung injury. The outflow of iron from FOXO1^high^ neutrophils is taken up by oligodendrocytes, thereby reducing the number and thickness of myelin sheath, contributing to oligodendrocyte damage associated with depression.

Using both genetic approaches and pharmacological treatment targeting FOXO1^high^ neutrophils, we have determined the protective role of FOXO1 against TBI. It indicates that modulating FOXO1 activity in neutrophils could be beneficial in attenuating TBI-induced brain damage and depression, paving the way for future therapeutic development. However, it is important to note that alternative explanations or other factors may have contributed to the observed effects. Previous studies have reported on various FOXO1-regulated signal pathways in brain tissue or microglia, including TrkB/PI3K/Akt/FOXO1 pathway, PTEN/PI3K/Akt/FOXO1/TLR4 pathway and PPARs/FOXO1/NLRP3 pathway. These pathways have been shown to increase neuroinflammation after cerebral hemorrhage, ischemic stroke, and psychological stress [57–59]. Nevertheless, there are limited and conflicting studies on the role of FOXO1 in microglia during TBI. Zhao et al. [60] showed that FOXO1 upregulated charged multivesicular body protein 4B to alleviate necroptosis of microglial induced by TBI, while Song et al. [61] reported that hesperetin improves neurobehavioral function after TBI by inhibiting inflammation response mediated by microglial activation via the AMPK-SIRT1-FOXO1-NF-κB axis. Regarding neutrophils, a previous study has shown that FOXO1 can induce C‐X‐C motif chemokine ligand 2 and C‐X‐C motif chemokine receptor 2 in bacterial infection that is associated with enhanced neutrophil mobilization. Additionally, FOXO1-induced integrins CD11b and CD18 can promote neutrophil migration as well as phagocytosis and bacterial killing [62]. Therefore, we hypothesize that FOXO1-regulated microglia may potentially exacerbate brain damage and depression post-TBI while PI3K/Akt, TLR/NF-κB or AMPK/SIRT1 signaling pathways may interact with FOXO1 to regulate neutrophil activities in TBI requiring further investigation. Furthermore, the relative specificity of these FOXO1^high^ neutrophils in TBI was largely confirmed by comparative analysis with other models of brain injuries, such as the mouse model of Alzheimer’s disease. However, it cannot be ruled out that FOXO1^high^ neutrophils may also be present in other types of brain injuries, such as stroke. This would strengthen the specificity of FOXO1^high^ neutrophil in TBI. Otherwise, it will expand the potential clinical significance of FOXO1^high^ neutrophils in various types of brain injuries and provide more space for exploration in terms of FOXO1^high^ neutrophil-related brain injuries or diseases.

In summary, we have identified novel functions of neutrophils post-TBI, highlighting their multifaceted capabilities. While further investigation is required to understand why FOXO1 is upregulated and why FOXO1^high^ neutrophils continue to infiltrate brain tissue, our findings shed light on the essential role of FOXO1^high^ neutrophils in TBI. Moreover, we demonstrated that targeted depletion of FOXO1^high^ neutrophils effectively attenuated acute and chronic brain damage and its consequences in mice with TBI. To date, randomized clinical trials targeting neutrophils have not shown improvement in TBI outcomes [63, 64]. The lack of success in these trials may be attributed partly to the phenotypic and functional heterogeneity of neutrophils [38, 65, 66]. Therefore, our results provide valuable insights into the function of neutrophils in TBI and offer new evidence for potential therapeutic strategies for TBI.

## Conclusions

Our fundings reveal a novel neutrophil phenotype characterized by elevated FOXO1 expression, which significantly exacerbates brain injury during both the acute and chronic stages of TBI. This discovery enhances our comprehension of the heterogeneity, reprogramming potential, and multi-functions exhibited by neutrophils in TBI, while also providing innovative evidence and therapeutic strategies for regulating neutrophil-related immune responses in TBI.

## Supplementary Information

**Additional file 1**: Materials and methods.** Table S1** siRNA sequences for the target genes.** Table S2** Specific primers for qRT-PCR analysis.** Table S3** CHIP primers for promoter sequences. **Fig. S1** A flowchart of the overall experimental design.** Fig. S2** The expression of FOXO1 in neutrophils was positively related to the injury degree of TBI in mice.** Fig. S3** Interfering with FOXO1 in neutrophils alleviates neurological deficits and reduces neuronal damage in the acute stage of TBI.** Fig. S4** FOXO1 regulates VCAN to delay neutrophil apoptosis.** Fig. S5** FOXO1 regulates the metabolism of neutrophils.** Fig. S6** FOXO1^high^neutrophils contribute to chronic damage of the brain post-TBI.** Fig. S7** KEGG enrichment analysis of proteomics in mice.

## Data Availability

The single cell-seq data involved in this study has been uploaded to the Genome Sequence Archive for Human China (https://ngdc.cncb.ac.cn/gsa-human), and could be retrieved by accession number HRA001915.

## Abbreviations

BCL-2: B-cell lymphoma-2
BAX: BCL-2-associated X protein
BBB: Blood-brain barrier
ChIP: Chromatin immunoprecipitation
Co-IP: Co-immunoprecipitation
FACS: Fluorescence-activated cell sorting
FBS: Foetal bovine serum
FOXO1: Forkhead box-proteinO1
FTH1: Ferritin heavy chain 1
FTL1: Ferritin light chain 1
GO: Gene ontology
IL: Interleukin
LC-MS: Liquid chromatography-mass spectrometry
LPO: Lipid hydroperoxide
MBP: Myelin basic protein
NeuN: Neuronal nuclear antigen
NK: Natural killer
OPCs: Oligodendrocyte progenitor cells
PBS: Phosphate-buffered saline
PDGF: Platelet-derived growth factor
qRT‑PCR: Quantitative real‑time PCR
scRNA-seq: Single-cell RNA sequencing
TBI: Traumatic brain injury
TFRC: Transferrin receptor
TNF: Tumor necrosis factor
VCAN: Versican
WT: Wild-type
xCT: System xc(−) cystine/glutamate antiporter
