# Supplementary material for: FOXO1 reshapes neutrophils to aggravate acute brain damage and promote late depression after traumatic brain injury
Source: Mil Med Res. 2024 Mar 31;11:20. doi: 10.1186/s40779-024-00523-w (PMC10981823; doi:10.1186/s40779-024-00523-w)
Supplement: Supplementary file 1 — Additional file 1: Materials and methods. Table S1 siRNA sequences for the target genes. Table S2 Specific primers for qRT-PCR analysis. Table S3 CHIP primers for promoter sequences. Fig. S1 A flowchart of the overall experimental design. Fig. S2 The expression of FOXO1 in neutrophils was positively related to the injury degree of TBI in mice. Fig. S3 Interfering with FOXO1 in neutrophils alleviates neurological deficits and reduces neuronal damage in the acute stage of TBI. Fig. S4 FOXO1 regulates VCAN to delay neutrophil apoptosis. Fig. S5 FOXO1 regulates the metabolism of neutrophils. Fig. S6 FOXO1highneutrophils contribute to chronic damage of the brain post-TBI. Fig. S7 KEGG enrichment analysis of proteomics in mice. [file 40779_2024_523_MOESM1_ESM.pdf]

Materials and methods

Experimental design

In the murine TBI model, we performed the behavioral and biological analyses at 48 h post-TBI (acute stage) and on day 30 post-TBI (chronic stage). FOXO1$^{\triangle Lyz2}$ mice and their wild-type littermate were randomly divided into sham group and TBI group, $n \geq 5$ mice in each group, respectively. Assays of the mortality and the behavioral indexes in mice were performed with 15 – 100 mice per group. The accurate number for every data collection and analysis of humans and mice was indicated in the involved figure legends. Each experiment was repeated three or more times.

Neurological deficit scores and behavioral tests

As described previously, the Longa score and a modified neurological severity score were used to assess the neurological deficits in each mouse on days 1 and 2 after TBI, which included motor, sensory function, reflex, balance beam, and foot faults [1]. The foot faults were recorded over 50 steps while the mice walked a narrow wooden beam 150 mm wide and 75 cm long. All mice were tested by two observers blinded to the treatment.

The sucrose preference was tested and used to assess the depression-like behavior of mice [2]. All the mice were free to choose drinking bottles between water and 1% sucrose solution without any stress. After 24 h, the location of the two bottles was changed. The fluid content drank by the mice was calculated. Sucrose preference (%) = sucrose intake/total fluid intake (water + sucrose intake) ×100%. The original body weight of each mouse was tested before constructing the TBI model, and the final weight was tested before testing depression-like behavior 1 month after TBI.

The tail suspension test was used to record the total immobility time to assess depression-like behavior [3]. Each mouse was taken from the cage and suspended from a steel beam (1 m in height) by adhesive tape. The mouse tail was taped approximately 3 cm from the tip. Every test session was recorded for 5 min. The test was monitored by a video surveillance system.

Dual-luciferase reporter assay

The HEK293 cells were obtained from the American Type Culture Collection (ATCC), and cultured in DMEM supplemented with 10% (v/v) FBS and antibiotics (100 U/ml penicillin and 100 μg/ml streptomycin) at a temperature of 37 °C in a 5% CO_2 atmosphere. The HEK293 cells were transiently transfected with reporter plasmids (pGFs) using Lipofectamine 3000 (L3000008, Thermo Fisher, USA). After 36 h of culture, the cell lysates were collected, and luciferase activity was measured with a dual luciferase reporter system according to the manufacturer's instructions (E1910, Promega, USA). The transfection experiments were repeated three times in triplicate, and the transfection efficiency was normalized by dividing firefly luciferase activity by Renilla luciferase activity.

Chromatin immunoprecipitation (ChIP)

ChIP was carried out using a Pierce Magnetic ChIP kit (26157, Thermo Scientific, USA) according to the manufacturer's protocol. Briefly, an equal amount (10 mg) of rabbit anti-FOXO1 (2880s, Cell Signaling Technology, USA) or normal rabbit IgG antibody (sc-2357, Santa Cruz, USA) was used to precipitate the cross-linked DNA/protein complexes of 5×10^7 mouse neutrophils. Following the reversal of cross-linking, the amount of chromatin precipitated by the indicated antibody was detected by polymerase chain reaction (PCR). The primers were listed in **Additional file 1: Table S3**.

Immunofluorescence staining

The method has been described previously [4]. In brief, cryostat sections (8 μm) were preincubated with a blocking buffer (PBS containing 2% BSA and 0.5% Triton-X100) for 30 min. For immunofluorescence staining, the cryostat sections were fixed with 4% paraformaldehyde for 30 min and then permeabilized with blocking buffer and blocked. The sections or coverslips were processed for immunostaining by incubating them with the primary antibodies overnight at 4 °C, followed by incubation with Alexa Fluor-conjugated secondary IgG antibodies for 1.5 h at room temperature. Subsequently, the sections or coverslips were washed, mounted, and observed under a confocal microscope. Primary antibodies against the following proteins were used: fibrinogen (1:200, ab58207,

Abcam, USA), FOXO1 (1:200, 2880s, Cell Signaling Technology, USA), myelin basic protein (MBP, 1:200, PA1-10008, Thermo Scientific, USA), and neuronal nuclear antigen (NeuN, 1:200, 36662, Cell Signaling Technology, USA), Versican (VCAN, 1:200, ET7107-09, Huabio, China), BAX (1:200, ET1603-34, Huabio, China), BCL-2 (1:200, ER0602, Huabio, China). Fluorescent images were captured using a fluorescence microscope (Olympus, VS200, Shinjuku, Tokyo) with excitation wavelengths appropriate for Alexa Fluor 350 (380 nm), 488 (488 nm), 594 (568 nm), 647 (628 nm).

Transmission electron microscopy

Brain blocks of less than 1 mm^3 in size and neutrophils of bone marrow were fixed in phosphate buffer containing 2.5% glutaraldehyde overnight and postfixed with 1% OsO_4 in phosphate buffer for 2 h. The samples were dehydrated and infiltration by a graded series of ethanol and acetone. The specimens were sectioned by LEICA EM UC7 ultratome. Finally, images were captured with a transmission electron microscope (Hitachi, HT7800). The inner and outer diameters of the myelin sheath were calculated with ImageJ.

Enzyme-linked immunosorbent assay (ELISA)

Million neutrophils were prepared to examine the levels of IL-1β, IL-6, and TNF-α. The kits (E-EL-M0037c, E-EL-MOO44C, E-MSEL-M0002, Elabscience) were used according to the manufacturer's instructions.

References

1. Yang T, Liu YWY, Zhao L, Wang H, Yang N, Dai SS, et al. Metabotropic glutamate receptor 5 deficiency inhibits neutrophil infiltration after traumatic brain injury in mice. Sci Rep. 2017;7(1):9998.
2. Zhou M, Liu Z, Yu J, Li SM, Tang M, Zeng L, et al. Quantitative proteomic analysis reveals synaptic dysfunction in the amygdala of rats susceptible to chronic mild stress. Neuroscience. 2018;376:24-39.
3. Liu YWY, Zhao L, Zhou M, Wang H, Yang N, Dai SS. Transplantation with mGluR5 deficiency bone marrow displays antidepressant-like effect in C57BL/6J mice. Brain Behav Immun. 2019;79:114-24.
4. Liu YW, Zhang JY, Bi WD, Zhou M, Li JB, Xiong TT, et al. Histones of neutrophil extracellular traps induce CD11b expression in brain pericytes via dectin-1 after traumatic brain injury. Neurosci Bull. 2022;38(10):1199-214.

Table S1 siRNA sequences for the target genes

Target genes	Forward primer (5' – 3')	Reverse primer (5' – 3')
FOXO1	GGAUUGAACCAGUAUAACUTT	AGUUAUACUGGUUCAAUCCTT
VCAN	CGACUGUUGGAGAACUUCATT	UUCACUCGUGUUGUAAUUGTT
TFRC	CCAGACCGUUAUGUUGUAGUA	UACUACAACAUAACGGUCUGG

FOXO1 forkhead box O1, *VCAN* Versican, *TFRC* transferrin receptor

Table S2 Specific primers for qRT-PCR analysis

Gene name	Forward primer (5' – 3')	Reverse primer (5' – 3')
FOXO1 (human)	GAGATCGACCCGGACTTCG	AGTTGGACTGGCTAAACTCCG
VCAN (human)	CACTCTAATCCCTGTCGT	ATGTCTCGGTATCTTGCT
CDKN1C (human)	GTCCCTCCGCAGCACATC	TCTGGTCCTCGGCGTTCA
FCGR3A (human)	GCAAAGGCAGGAAGTATT	GTACCCAGGTGGAAAGAA
GNLY (human)	AGAAATCCTGCCCGTGCCT	TCGTGACCTCCCCGTCCT
CCL5 (human)	TGCTTTGCCTACATTGCC	TGACCTGTGGACGACTGC
PLAUR (human)	CACTCACGAACGCTCACT	TAGCTTGGGCTTCCTCAC
CD247 (human)	AAAGCCGAGAAGGAAGAA	GAAGGGCGTCGTAGGTGT
NEAT1 (human)	GACCAAGGGCTGTGAACC	CACCCAGACCTGGACGCT
FOXO1 (mouse)	AGTGGATGGTGAAGAGCGTG	GAAGGGACAGATTGTGGCGA
VCAN (mouse)	AGGGGTTCGGACCTATGG	TGGGAGCAGTGATGTGGA
FCGR3A (mouse)	TAACGAAAGCCTCATCCC	AGCAATAGCCAGCCCATA
RPL3 (mouse)	ATGCGTCTGCTTCCTCTA	GTACCCTTTGCCTTTTGT
CD247 (mouse)	AAGTGGAAAGTGTCTGTT	TGACTCCGTAGATGAAGA
NEAT1 (mouse)	ATGTCTTGTTCTGGGAGC	CCTTACGCAATCTTCTCG
HCST (mouse)	GCTGCAAGTCAGACATCG	GCGGACAACAGTAGTAGC

FOXO1 forkhead box O1, *VCAN* Versican, *CDKN1C* cyclin-dependent kinase inhibitor 1C, *FCGR3A* Fc gamma receptor IIIa, *GNLY* granulysin, *CCL5* C-C motif chemokine ligand 5, *PLAUR* plasminogen activator, urokinase receptor, *CD247* T-cell receptor T3 zeta chain, *NEAT1* nuclear paraspeckle assembly transcript 1, *RPL3* ribosomal protein L3, *HCST* hematopoietic cell signal transducer

Table S3 ChIP primers for promoter sequences

Promoter region	Forward primer (5' – 3')	Reverse primer (5' – 3')
VCAN	TTTGCTTCTGCCCTACTTC	GGTTTGCCCAAGGCTACT
TFRC	TCTCCCTCCTCCTCTTCC	GGCCAGCCTGGTCTACAT

ChIP chromatin immunoprecipitation, *VCAN* Versican, *TFRC* transferrin receptor

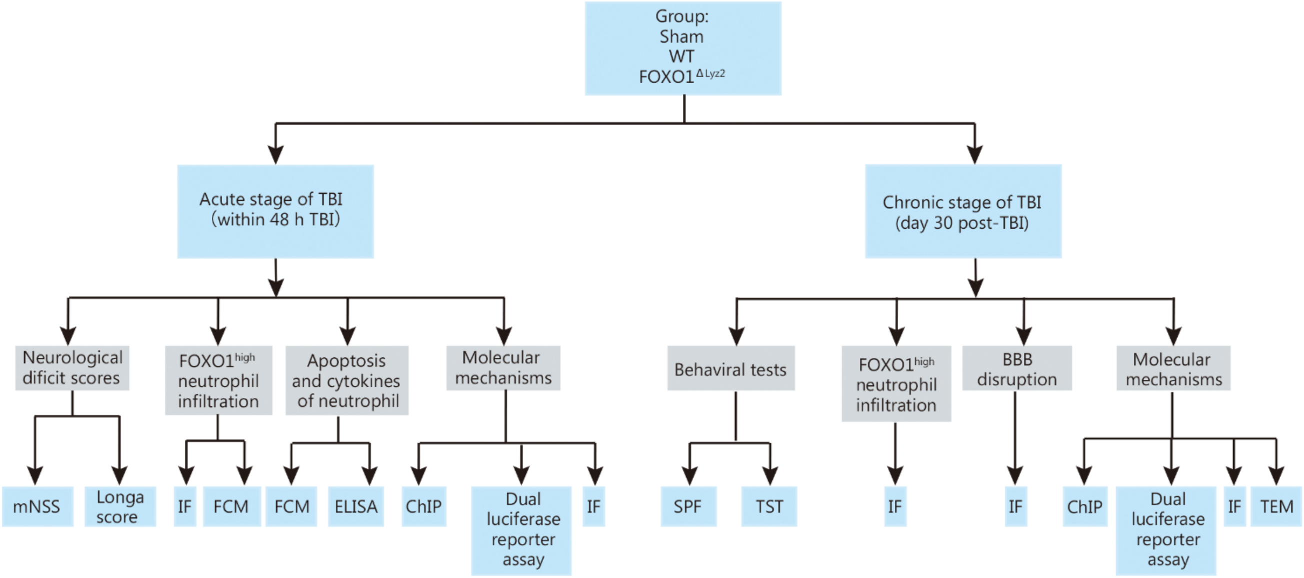

Fig. S1 A flowchart of the overall experimental design. The chronological order of behavioral and biological analyses was performed at 48 h post-TBI (acute stage) and on day 30 post-TBI (chronic stage). mNSS modified neurological severity score, IF immunofluorescence staining, FCM flow cytometry, ELISA enzyme-linked immunosorbent assay, ChIP chromatin immunoprecipitation, SPF sucrose preference test, TST tail suspension test, TEM transmission electron microscopy, TBI traumatic brain injury, BBB blood-brain barrier, FOXO1 forkhead box protein O1

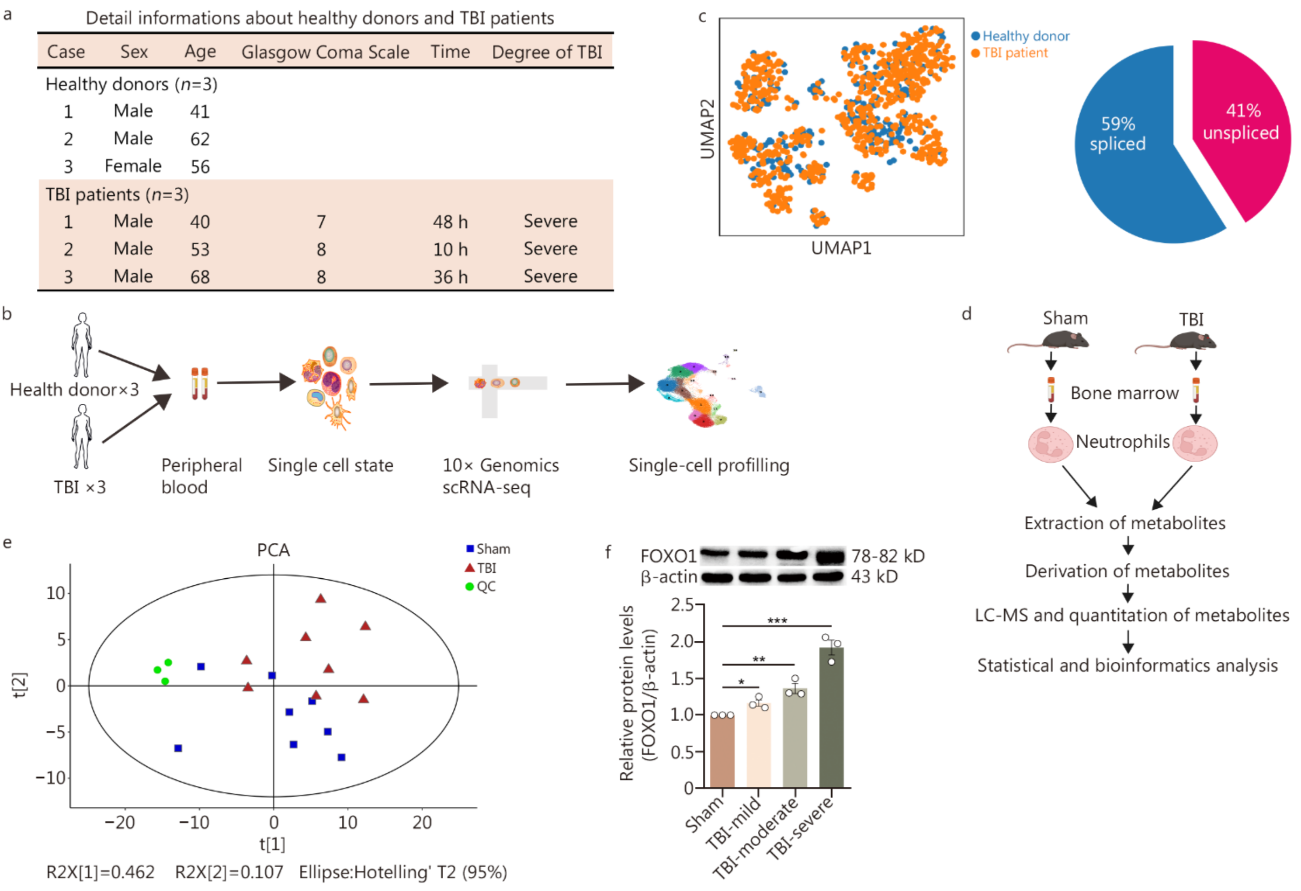

Fig. S2 The expression of FOXO1 in neutrophils was positively related to the injury degree of TBI in mice. **a** Detailed information on human samples. TBI patients are labeled in red letters, and healthy donors are labeled in blue. **b** Graphical scheme describing the experimental workflow of the single-cell sequencing study. **c** UMAP plot depicting the distribution of gene expression between the healthy donors and TBI groups. Pie chart showing the proportion in different groups; blue represents spliced, and red represents unspliced. **d** Graphical scheme describing the experimental workflow for metabolomics. **e** Scatter plot showing the quality control of metabolomics in each group. **f** Western blotting showing the expressions of FOXO1 in neutrophils from the sham group and the mice with different degrees of TBI (n = 5 – 10 per group). The experiment was repeated three times and data are represented as the mean ± SEM. $^{*}P < 0.05$, $^{**}P < 0.01$, $^{***}P < 0.001$. FOXO1 forkhead box protein O1, TBI traumatic brain injury, UMAP uniform manifold approximation, and projection, scRNA-seq Single-cell RNA sequencing, QC quality control, LC-MS Liquid chromatograph mass spectrometer

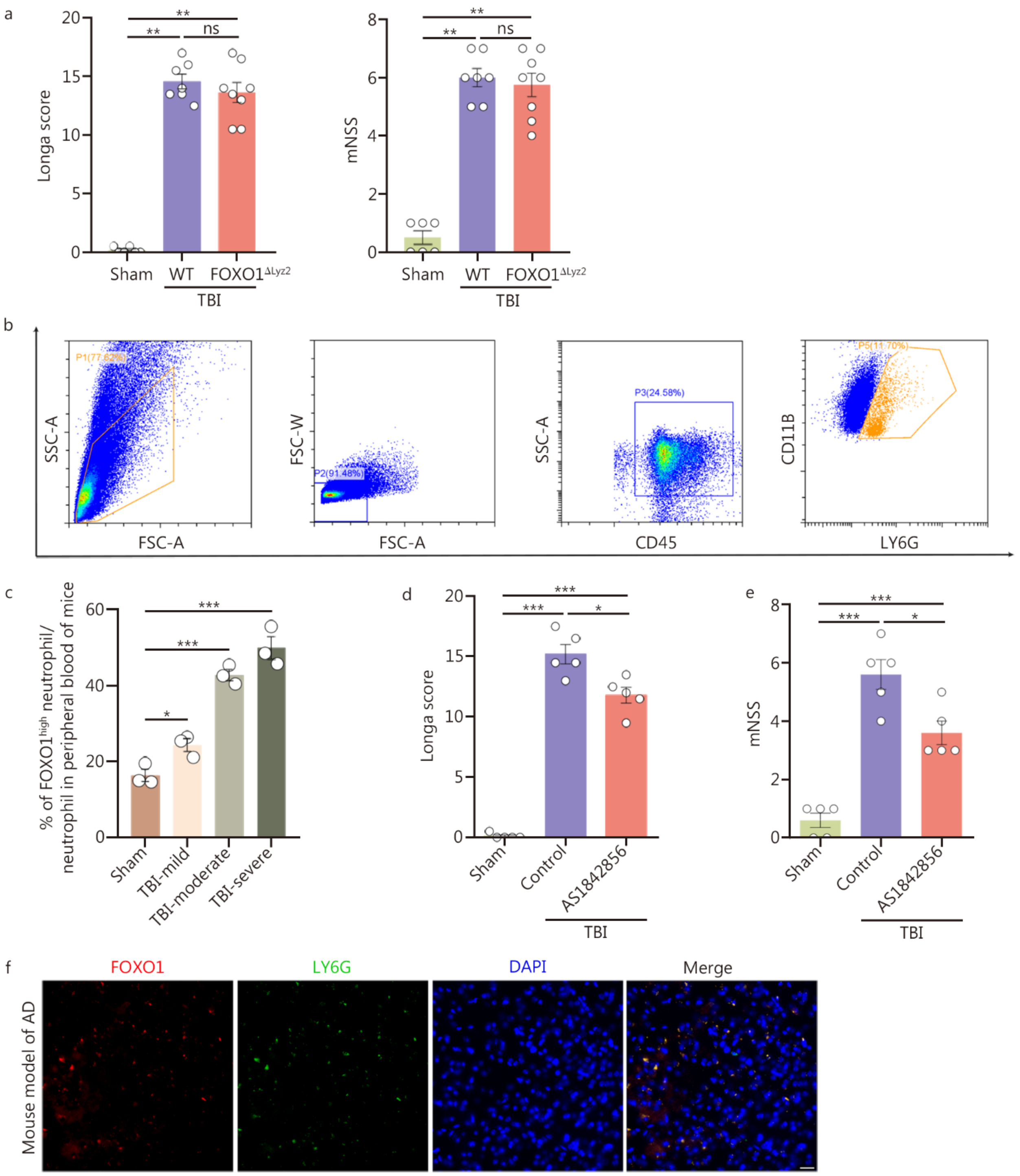

Fig. S3 Interfering with FOXO1 in neutrophils alleviated neurological deficits and reduced neuronal damage in the acute stage of TBI. **a** Behavioral recovery of mice was assessed using the Longa score and mNSS at 24 h post-TBI. Sham group: *n* = 6; WT TBI mice: *n* = 7; FOXO1$^{\triangle Lyz2}$ TBI mice, *n* = 8. **b** Flow cytometry analysis of the percentage of $CD45^{+}CD11b^{+}$ neutrophils among $CD45^{+}$ cells in peripheral blood. The experiment was performed three times. **c** Flow cytometry analysis of the percentage of FOXO1^{high} neutrophils in total circulating neutrophils in mice with different degrees of TBI (*n* = 5). **d** Behavioural recovery of TBI mice treated with 10 mg/kg FOXO1 inhibitor AS1842856 via intraperitoneal injection was assessed by the Longa score 24 h post-TBI (*n* = 5). **e** Behavioural

recovery of TBI mice treated with 10 mg/kg FOXO1 inhibitor AS1842856 via intraperitoneal injection was assessed by the mNSS 24 h post-TBI ($n = 5$). **f** Immunostaining of $FOXO1^{high}$ neutrophils in brain tissues from a mouse model of AD induced in transgenic APP/PS1 mice. Markers: LY6G (green), FOXO1 (red), and nuclei (blue). Scale bar = 10 μm. Data are represented as the mean ± SEM. $^{*}P < 0.05$, $^{**}P < 0.01$, $^{***}P < 0.001$, ns non-significant. FOXO1 forkhead box protein O1, TBI traumatic brain injury, mNSS modified neurological severity score, DAPI 4,6-diamidino-2-phenylindole dihydrochloride

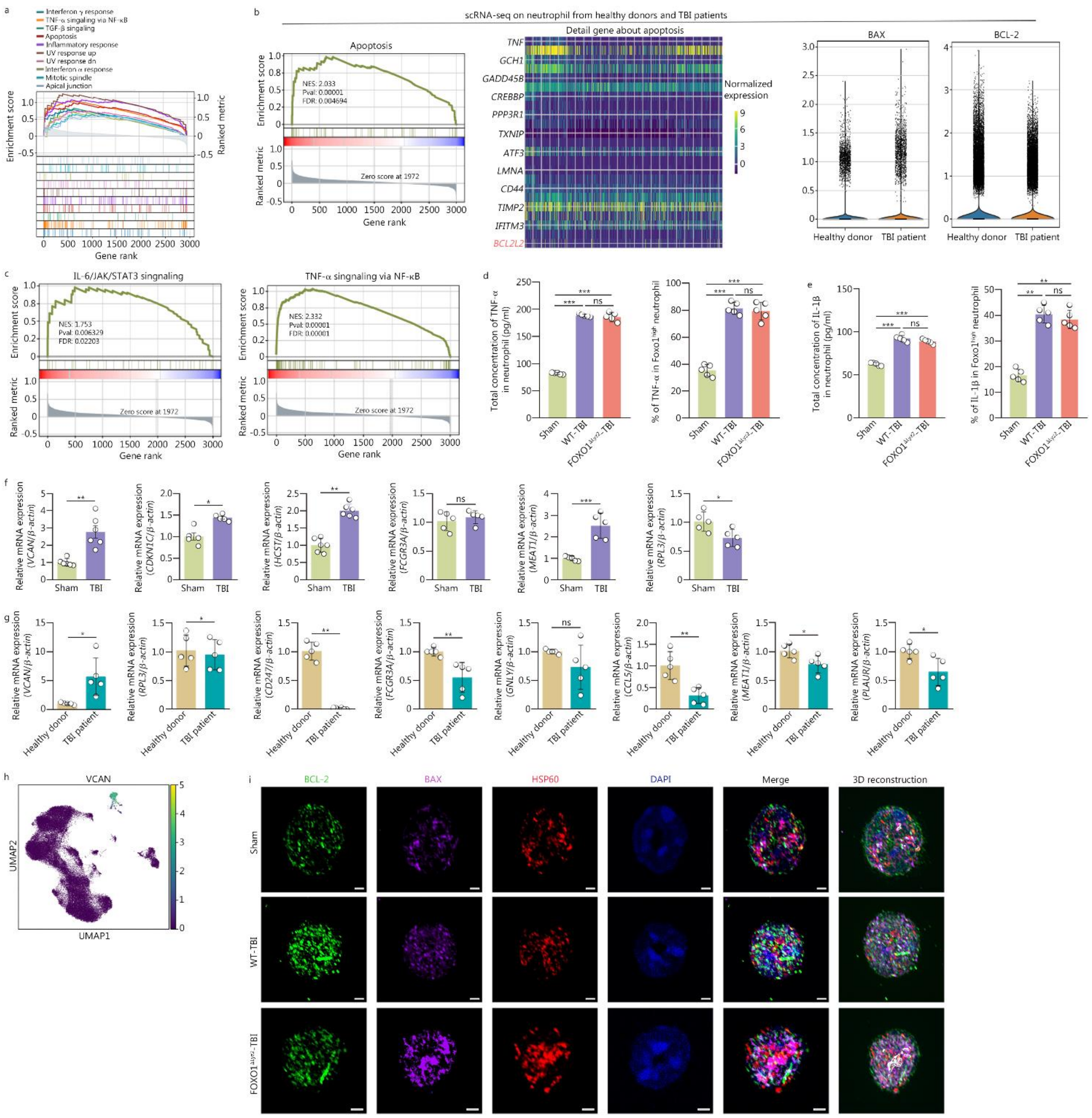

Fig. S4 FOXO1 regulates VCAN to delay neutrophil apoptosis. **a** Gene set enrichment analysis (GSEA) plot showing the enrichment scores for total pathways. **b** GSEA showing the apoptosis pathway and the detailed genes in neutrophils. Pathway enrichment analysis was performed based on scRNA-seq data of neutrophils from patients and healthy donors. **c** GSEA plot showing the enrichment scores for the TNF-α and IL-6 pathways in neutrophils. Pathway enrichment analysis was performed based on scRNA-seq data of neutrophils from patients and healthy donors. **d** ELISA analysis (left) and flow cytometry (right) of TNF-α levels in neutrophils from mice ($n = 5$). **e** ELISA analysis (left) and flow cytometry (right) of IL-1β levels in neutrophils from mice ($n = 5$). **f** The difference of target gene mRNA expressions in murine neutrophils between the sham and TBI group ($n = 5$). **g** Bar plots showing the target gene mRNA expression in neutrophils from TBI patients. Healthy donors served as control. $n = 5$ for each group. **h**

UMAP plot depicting the expression of VCAN in neutrophils by scRNA-seq. **i** Immunostaining showing the expression, location, binds, and 3D reconstruction between BAX and BCL-2 in neutrophils of mice (n = 5). Mitochondria marker BCL-2 (green), BAX (purple), HSP60 (red), and nuclei (DAPI, blue). Scale bar =1 μm. **a-c, h** scRNA-seq data analysis of human peripheral blood neutrophils (TBI patients vs. healthy donors). Each experiment was repeated three times. Data are represented as the mean ± SEM. $^{*}P < 0.05$, $^{**}P < 0.01$, $^{***}P < 0.001$, ns non-significant. FOXO1 forkhead box protein O1, VCAN Versican, TBI traumatic brain injury, WT wild-type, scRNA-seq single-cell RNA sequencing, ELISA enzyme-linked immunosorbent assay, HSP60 heat shock protein 60, BCL-2 B-cell lymphoma-2, BAX BCL-2-associated X protein, DAPI 4,6-diamidino-2-phenylindole dihydrochloride, UMAP uniform manifold approximation, and projection

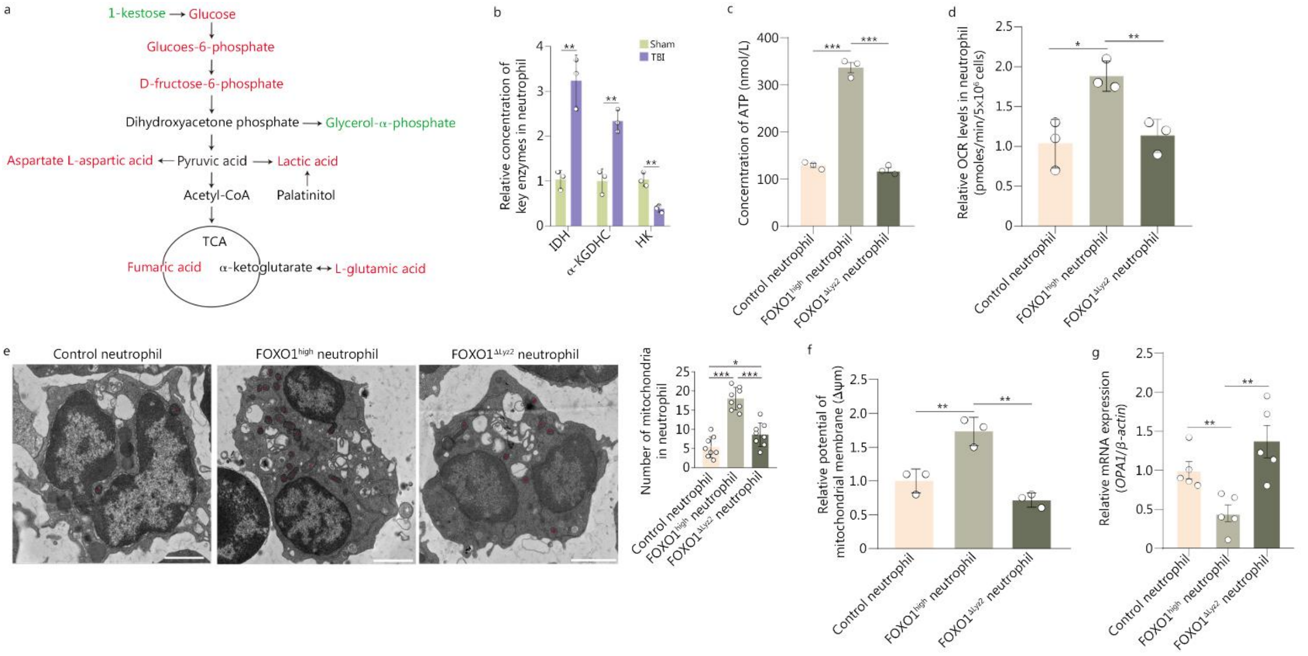

Fig. S5 FOXO1 regulates the metabolism of neutrophils. **a** Detailed metabolites of neutrophils from mice associated with glycolysis and the TCA cycle. TBI group vs. sham group, red, indicates an increase; green, indicates a decrease. **b** ELISA analysis of key enzymes of glycolysis/TCA in neutrophils of sham group and TBI group (n = 5). The experiment was repeated three times. **c** The generation of ATP in neutrophils was detected by the ATP Assay Kit. **d** The OCR was detected as an indicator for OXPHOS and deduced levels of basal respiration and ATP production. **e** Electron microscopy image of the mitochondrial form in neutrophils. Scale bar = 2 μm. **f** The MMP was detected by the JC-1 Assay Kit. **g** Bar plots showing the mRNA expression of *OPA1* in neutrophils. In **c** and **d**, normal neutrophils of mice served as the control, FOXO1 ^{high} neutrophils and FOXO1$^{\triangle Lyz2}$ neutrophils were used to detect the effects of FOXO1 on these neutrophil activities. Each experiment was repeated three times and each group was assayed in triplicate. Data are represented as the mean ± SEM. $^{*}P < 0.05$, $^{**}P < 0.01$, $^{***}P < 0.001$. FOXO1 forkhead box protein O1, TCA tricarboxylic acid cycle, ELISA enzyme-linked immunosorbent assay, OCR oxygen consumption rate, OXPHOS oxidative phosphorylation, OPA1 optic atrophy 1, MMP mitochondrial membrane potential, IDH Isocitrate dehydrogenase, α-KGDHC α-ketoglutarate dehydrogenase complex, HK hexokinase

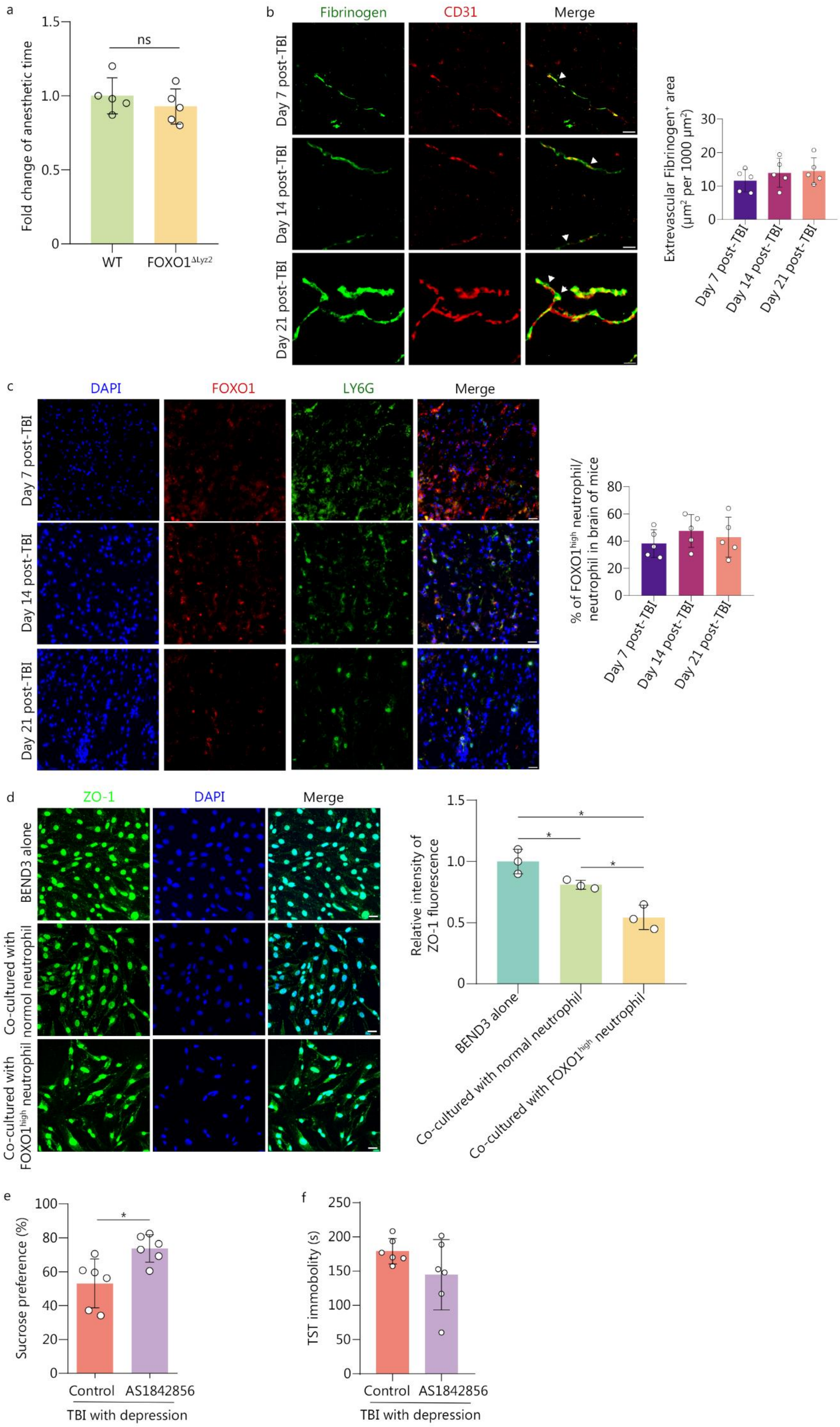
a
Fold change of anesthetic time
ns
WT
FOXO1ΔLyz2
b
Fibrinogen
CD31
Merge
Day 7 post-TBI
Day 14 post-TBI
Day 21 post-TBI
Extravascular Fibrinogen+ area (μm2 per 1000 μm2)
c
DAPI
FOXO1
LY6G
Merge
% of FOXO1high neutrophil/ neutrophil in brain of mice
d
ZO-1
DAPI
Merge
BEND3 alone
Co-cultured with normol neutrophil
Co-cultured with FOXO1high neutrophil
Relative intensity of ZO-1 fluorescence
Co-cultured with normal neutrophil
e
Sucrose preference (%)
Control
AS1842856
TBI with depression
f
TST immobility (s)

Fig. S6 FOXO1^{high} neutrophils contribute to chronic damage of the brain post-TBI. **a** The anesthetic recovery time of mice after TBI ($n = 5$). **b** Dynamic BBB disruption of mice on day 7, day 14, and day 21 post-TBI. Immunostaining of fibrinogen (green) and CD31 (red) in the brain tissues of TBI mice. Scale bar = 20 μm. Five mice at each time point were enrolled in each group. **c** Dynamic infiltration of FOXO1^{high} neutrophils in brain tissues of mice on day 7, day 14, and day 21 post-TBI. Immunostaining of FOXO1 (red), LY6G (green), and nuclei (DAPI, blue) in brain injury tissue of the TBI mouse model. Five mice at each time point were enrolled in each group. **d** Effect of FOXO1^{high} neutrophils on zonula occludens-1 (ZO-1) expressions in brain microvascular endothelial cells. Immunostaining of ZO-1 (green) and nuclei (DAPI, blue) in brain microvascular endothelial cells BEND3. Each experiment was repeated three times and each group was assayed in triplicate. **e** Behavioural test of the sucrose preference. **f** Behavioural tests of tail suspension test (TST). In **e** and **f** the mice with TBI-induced depression were treated with 10 mg/kg FOXO1 inhibitor AS1842856. The mice vehicle treatment served as the control group. The indexes were assayed at 1 d after intraperitoneal injection, $n = 6$ in each group. $^{*}P < 0.05$, ns non-significant. FOXO1 forkhead box protein O1, TBI traumatic brain injury, BBB blood-brain barrier, DAPI 4,6-diamidino-2-phenylindole dihydrochloride, BEND3 brain-derived endothelial cells 3

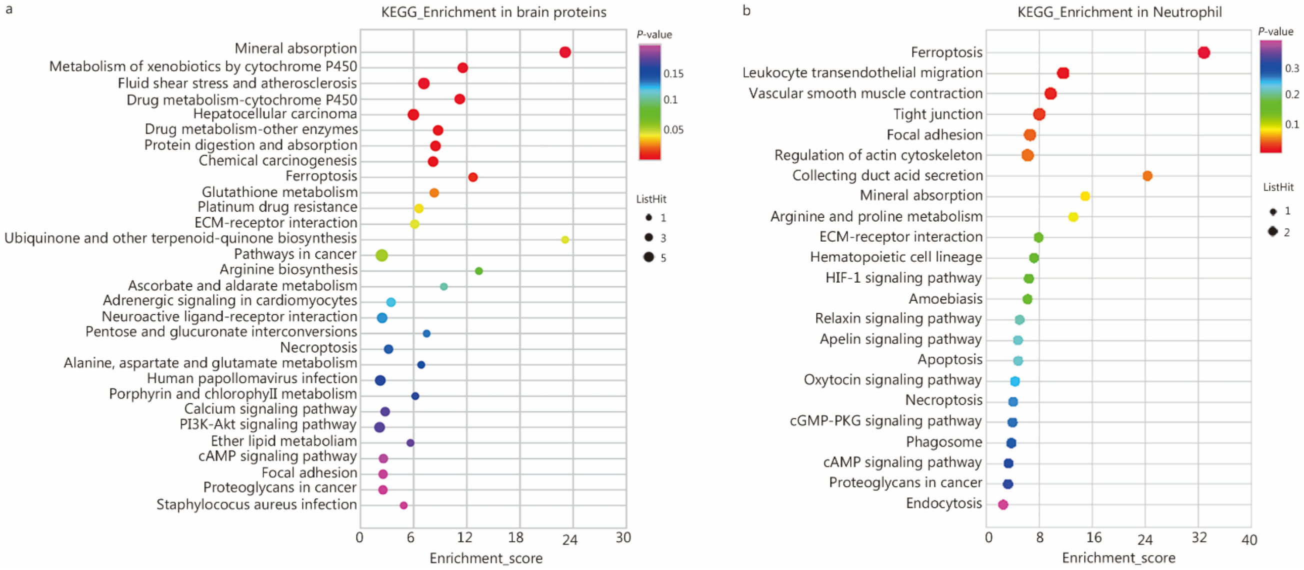

Fig. S7 KEGG enrichment analysis of proteomics in mice. **a** KEGG enrichment analysis of murine brain tissue proteomics (TBI with depression group vs. TBI without depression group). **b** KEGG enrichment analysis of murine bone marrow neutrophil proteomics (TBI with depression group vs. TBI without depression group). KEGG Kyoto Encyclopedia of Genes and Genomes, TBI traumatic brain injury, ECM extra cellular matrix, PI3K phosphoinositide 3-kinase, Akt protein kinase B, cAMP cyclic adenosine monophosphate, HIF-1 targeting hypoxia-inducible factor 1, cGMP cyclic guanosine monophosphate, PKG protein kinase G
